# CAVE-Onc: Graph-constrained agentic validation for cross-domain contradictions in CDISC oncology submissions

**DOI:** 10.1371/journal.pone.0350376

**Published:** 2026-08-14

**Authors:** Jaime Yan

**Affiliations:** Harrisburg University of Science and Technology, Harrisburg, Pennsylvania, United States of America; Sichuan University, CHINA

## Abstract

The CDISC Rules Engine (CORE) provides industry-standard validation for Study Data Tabulation Model (SDTM) submissions, yet its imperative, domain-scoped architecture cannot express cross-domain contradictions—such as a Response Evaluation Criteria in Solid Tumors (RECIST) 1.1 overall response that contradicts the combined target lesion, non-target lesion, and new-lesion status—which pass structural validators undetected. We present CAVE-Onc, a two-layer graph-constrained agentic validation engine that augments CORE with declarative Shapes Constraint Language (SHACL) validation (L1) and a LangGraph-based agentic orchestrator (L3). CAVE-Onc operates over an RDF knowledge graph built from XPT datasets. Layer 1 comprises 111 SHACL shapes—85 ported from CORE rules, 8 RECIST 1.1 derivation shapes, and 18 archetype-specific SHACL-SPARQL cross-domain constraint shapes—while Layer 3 implements a CaveAgent for RECIST Table 7 verification. We evaluated CAVE-Onc against pre-registered hypotheses (OSF): Track A assessed complementarity with CORE on clean data (52 subjects); Track B evaluated detection of 20 contradiction archetypes injected via a RELREC-preserving generator. On Track A, CAVE L1 and CORE produced disjoint flag sets (Jaccard = 0.004). On Track B, CAVE detected 20/20 archetypes (100%; 95% CI: 83.2%–100.0%): 19 via L1 SHACL-SPARQL constraints and 1 via L3 agent exclusively. Two industry validators—the CDISC CORE engine (8/20) and the Pinnacle 21 FDA engine (6/20)—each detected 0/10 of the cross-domain RECIST contradictions they cannot express (all detections were CORE-seeded structural checks), versus CAVE’s 10/10 (McNemar *p* = 0.002). Three domain experts validated all 20 archetypes (Fleiss’ κ=0.705; 0 rated invalid). Because the archetypes were authored knowing the injected patterns, we frame this as construction validation of *expressiveness*, not a real-world detection estimate; on five held-out archetypes, existing shapes detected 3/5. On two real oncology trials mapped to SDTM, the engine stayed specific on unmutated data and detected 10/11 and 16/18 of applicable archetypes. Graph-based validation thus augments rather than replaces industry-standard tools.

## Introduction

Clinical trial data submitted to regulatory authorities must conform to the Study Data Tabulation Model (SDTM) standards maintained by the Clinical Data Interchange Standards Consortium (CDISC) [1]. The CDISC Rules Engine (CORE) [2] provides an open-source, imperative validation framework that evaluates domain-scoped rules (field lengths, controlled terminology, required variables) encoded in YAML/JSON definitions sourced from the CDISC Library. Pinnacle 21 Community [3], the industry-standard validator used across pharmaceutical submissions, can execute the CORE engine (as of Community 4.0) alongside its own domain-scoped structural validation engines.

Despite its breadth, CORE operates within a fundamental expressiveness boundary. Rules execute as vectorized pandas operations over isolated domain DataFrames. Cross-domain constraints—those requiring joins across, say, Tumor Results (TR), Tumor Identification (TU), and Response (RS) domains to verify Response Evaluation Criteria in Solid Tumors (RECIST) 1.1 overall response consistency—fall outside this architecture. CORE rules can reference at most one domain or a pre-joined pair; they cannot traverse graph-shaped relationships or perform multi-step clinical reasoning. The consequence is not merely a coverage gap but a *structural* gap: certain categories of data contradictions are inexpressible in CORE’s rule language, regardless of rule authoring effort.

This limitation matters for oncology submissions governed by RECIST 1.1 criteria [4]. An overall response of “Complete Response” (CR) recorded in RS when target lesion shrinkage indicates Partial Response (PR), non-target disease is Stable Disease (SD), and no new lesions have appeared, represents a clinical contradiction that passes CORE and Pinnacle 21 without flagging. Such contradictions, if undetected, may compromise the data quality that regulatory submissions require under U.S. Food and Drug Administration (FDA) electronic submission standards [5].

We contribute CAVE-Onc, a two-layer graph-constrained agentic validation engine designed to detect cross-domain clinical contradictions in CDISC oncology submissions. CAVE-Onc combines: (1) a Layer 1 (L1) Shapes Constraint Language (SHACL) validation suite of 111 shapes—85 ported from the CORE oncology rule corpus with zero expressiveness compromise, plus 8 RECIST 1.1 derivation shapes and 18 archetype-specific SHACL-SPARQL constraint shapes—and (2) a Layer 3 (L3) LangGraph-based [6] agentic orchestrator that performs RECIST Table 7 overall response verification through structured tool calls over a Resource Description Framework (RDF) knowledge graph. A planned Layer 2 (probabilistic DAG) was dropped after Gate B analysis determined that only 1 of 20 enumerated contradiction archetypes required probabilistic reasoning, below the 10-archetype threshold for maintaining a separate layer.

We evaluate CAVE-Onc against pre-registered hypotheses on two tracks: Track A characterizes rule-class complementarity with CORE on clean reference data, and Track B measures contradiction detection on a synthetic corpus of 20 clinician-reviewed archetypes injected into open-source datasets [7]. All evaluation data, code, and the OSF pre-registration protocol are publicly available.

CDISC validation tools primarily emphasize conformance to structural rules, controlled terminology, and field-level consistency. CORE [2] provides an open-source execution engine for these checks and is the baseline most directly aligned with the validation problem addressed here. Complementary standards-oriented resources, including SENDConform [8] and graph-based metadata repositories [9], show the value of semantic representations for biomedical standards, but they do not provide an oncology-specific benchmark for cross-domain contradiction detection. Graph validation provides a natural substrate for expressing relationships that span SDTM domains. SHACL defines a standard constraint language for RDF graphs [10], and pySHACL provides mature support for SHACL-SPARQL (SPARQL Protocol and RDF Query Language) constraints in Python [11]. CAVE-Onc uses these capabilities to express multi-domain joins, temporal comparisons, conditional existence checks, and valueset cross-references over an RDF representation of SDTM data. RECIST 1.1 defines the clinical response logic needed for oncology efficacy assessment [4]. Existing validators can check many domain-local data-quality rules, but overall response verification requires combining target lesion measurements, non-target lesion assessments, and new-lesion status. CAVE-Onc addresses this gap by pairing declarative graph constraints with a constrained LangGraph agent [6] for the decision-matrix logic that is most awkward to maintain as a single monolithic query.

## Materials and methods

### Ethics statement

This study analyzed only publicly available, de-identified and synthetic datasets—the CDISC SDTM/ADaM Pilot Project data and the open-source pharmaversesdtm package [7]—and did not involve human participants, identifiable patient data, or the collection of new data. Accordingly, institutional review board (IRB) approval and informed consent were not applicable to this work.

### Two-layer architecture

**Fig 1:**
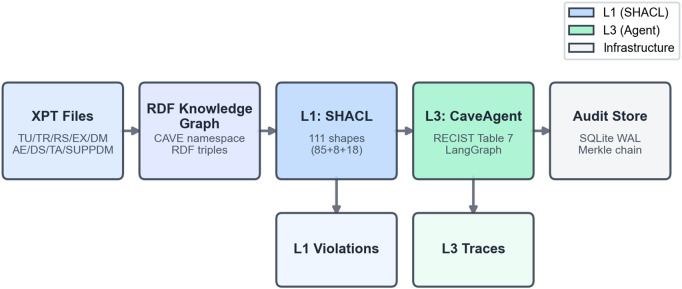
CAVE-Onc two-layer validation architecture. XPT files are converted to an RDF knowledge graph, validated by 111 SHACL shapes (L1), and selectively routed to a LangGraph CaveAgent (L3) for RECIST Table 7 reasoning. All traces are recorded in a Merkle-chained audit store.

The original design specified three layers: L1 (SHACL), L2 (probabilistic DAG), and L3 (agentic). After Gate B analysis determined that only 1 of 20 archetypes required multi-step reasoning beyond declarative graph constraints, L2 was dropped. The L1/L3 numbering is retained to match the pre-registration protocol. CAVE-Onc processes CDISC submission data through the following pipeline (Fig 1):

1. **XPT-to-RDF adapter.** SAS Transport (XPT) files for nine oncology domains (TU, TR, RS, EX, DM, AE, DS, TA, SUPPDM) are converted to RDF triples using a custom adapter that preserves variable labels, controlled terminology bindings, and RELREC foreign-key relationships under a dedicated CAVE namespace. The adapter handles trial-design domains without USUBJID (e.g., TA) via hash-based IRIs and expands SUPPDM qualifiers (QNAM/QVAL) into semantic cave:SUPP_{QNAM} triples for downstream cross-domain reasoning.
2. **Layer 1 (SHACL validation).** The RDF graph is validated against 111 SHACL shapes using pySHACL (v0.31) [10,11], an rdflib-backed Python SHACL validator. Shapes are partitioned into three categories: 85 CORE-ported shapes (see SHACL shape porting pipeline), 8 RECIST derivation shapes (see RECIST 1.1 derivation shapes), and 18 archetype-specific SHACL-SPARQL cross-domain constraint shapes (see Archetype-specific SHACL-SPARQL shapes). L1 produces a structured violation report as (USUBJID, shape_id, severity) tuples. Shapes targeting trial-design domains (TA) or supplemental qualifier domains (SUPPDM) produce flags without an associated USUBJID; these appear as “unknown” domain in the flag breakdown. Note: the original design specified Trav-SHACL over an Oxigraph triple store, but pySHACL was selected for its mature SHACL-SPARQL support; migration to Trav-SHACL is recommended for production scaling (see Discussion).
3. **Layer 3 (LangGraph agent).** Subjects with L1 violations or those flagged by routing heuristics enter the CaveAgent, a LangGraph state machine with typed Pydantic schemas. The agent has access to SPARQL query tools over the RDF graph and a RECIST Table 7 lookup table [4]. L3 invocation rate is a primary reported metric.
4. **Audit store.** All validation traces are written to an append-only SQLite WAL store with a Merkle hash chain linking consecutive entries, providing a tamper-evident audit trail foundation compatible with 21 Code of Federal Regulations (CFR) Part 11 requirements [12].

### SHACL shape porting pipeline

We systematically ported CORE rules scoped to oncology domains (TU, TR, RS, EX, DM) into SHACL shapes:

- **Source:** CORE v0.15 cache snapshot (commit 88837395), oncology-filtered to *n* = 122 rules.
- **Classification pipeline:** Rules were classified as single-domain (portable to SHACL) or cross-domain (unportable). Single-domain rules underwent polarity auditing and recursive De Morgan expansion.
- **Port success:** 85/122 rules (69.7%) ported with zero expressiveness compromise. The 37 unportable rules fell into two buckets: 31 cross-domain join rules and 6 row-set uniqueness rules.
- **Port-kind distribution:** De Morgan expansion (50/85, 58.8%), polarity flip (30/85, 35.3%), no change (5/85, 5.9%).
- **Per-domain breakdown:** DM = 74 shapes (67.9% port rate), EX = 5 (71.4%), TR = 4 (100%), RS = 2 (100%).

Full porting statistics, including per-rule classification and polarity-audit outcomes, are in S2 Table in S1 File.

### RECIST 1.1 derivation shapes

Eight SHACL-SPARQL shapes were authored from RECIST 1.1 clinical criteria [4] to validate derivation logic between TR (tumor measurements) and RS (response assessments). These shapes enforce target-lesion JOIN patterns, lymph-node short-axis VALUES, two-stage baseline sub-queries, and confirmation-window requirements. Shapes were reviewed and approved by an independent clinical reviewer.

### Archetype-specific SHACL-SPARQL shapes

Eighteen SHACL-SPARQL shapes were authored to target 18 of the 20 contradiction archetypes (A01–A15, A17, A18, A20) using cross-domain SPARQL constraints within sh:sparql blocks. Of the remaining two archetypes, A16 (duplicate USUBJID across studies) is a row-set uniqueness contradiction with no dedicated archetype shape (the duplicated record re-triggers the existing domain shapes, so it is credited structurally as a side-effect rather than by a bespoke cross-domain constraint), and A19 is handled by the L3 agent. These shapes implement nine distinct constraint patterns: tumor response consistency (A01, A06, A18), new lesion detection (A02), temporal consistency (A03, A07, A20), visit alignment (A04), supplemental qualifier cross-reference (A05, A13), valueset cross-reference (A08), conditional existence (A09, A12, A14), date derivation (A10, A11, A15), and cross-record consistency (A17).

### CaveAgent with RECIST Table 7 lookup

The L3 CaveAgent implements RECIST Table 7 overall response verification. Given a subject’s visit-level data across TR, RS, and TU domains, the agent: (1) queries the RDF graph for target lesion Sum of Longest Diameters (SLD) change from baseline; (2) queries non-target overall response; (3) queries new lesion status; (4) applies the RECIST Table 7 decision matrix to determine the expected overall response; (5) compares the expected response against RSORRES and emits a trace if they conflict. The agent operates within LangGraph’s constrained state machine, ensuring deterministic execution paths.

### Contradiction injection corpus

Twenty contradiction archetypes were enumerated with clinical review (Gate B) covering nine domains (RS, TR, TU, DM, EX, AE, DS, TA, SUPPDM); the full per-archetype catalog is in S1 Table in S1 File. By provenance the archetypes fall into two groups: ten (A08–A17) were derived during the Gate A gap analysis from CORE’s own conformance-rule corpus—each corresponds to an existing CORE rule that CAVE re-expresses as a cross-domain shape—while the other ten (A01–A07, A18–A20) are RECIST 1.1 overall-response and protocol-extension contradictions with no corresponding CORE rule. This split underlies the Track B baseline comparison (see Results). Each archetype was injected into clean pharmaversesdtm data [7] using a RELREC-preserving injector that modifies clinical values at the semantic layer while maintaining referential integrity.

### Evaluation design

Evaluation was pre-registered (OSF: https://osf.io/fx2ky) with four hypotheses (Table 1).

**Table 1:** Pre-registered evaluation hypotheses.

Hypothesis	Track	Metric	Threshold
H1: Rule-class complementarity	A	Jaccard(L1, CORE)	< 0.10
H2: CAVE novelty	B	CAVE-only catches	≥1 archetype
H3: Agent layer value	B	L3-only detection on A19	≥1 trace
H4: Runtime efficiency	B	Pipeline overhead ratio	≤10.0×

**Track A** (regression/complementarity): CDISC Pilot 1 data (52 subjects, oncology domains DM and EX) was validated by both CAVE L1 and CORE CLI. Flag sets were compared as (USUBJID, rule_id) tuples. Note: pharmaversesdtm RECIST data (6 subjects) was used for Track B injection only; Track A results are reported on Pilot 1.

**Track B** (novelty): Each of 20 archetypes was injected into the clean corpus. Four configurations were run: two empirical industry baselines (the Pinnacle 21 Community FDA production engine and the CORE engine), L1-only (ablation), and full CAVE (L1 + L3). Detection was measured as binary (detected/not detected) per archetype.

### Audit trail

All validation runs produce append-only traces in a SQLite WAL store. A Merkle hash chain links consecutive entries, enabling post-hoc tamper detection. This provides a foundation for 21 CFR Part 11 compliance [12]; full Part 11 implementation (e-signatures, RBAC, IQ/OQ/PQ) is out of scope.

## Results

### Track A: Rule-class complementarity

On clean reference data (52 subjects from CDISC Pilot 1, oncology domains DM and EX), CAVE L1 and CORE produced substantially different flag sets (Table 2).

**Table 2:** Track A flag comparison on clean data.

Metric	CAVE L1	CORE	Overlap
Total flags	5,803	941	24
DM domain	5,108	343	—
EX domain	591	598	—
Unknown	104	0	—
Unique rules	—	18	—

The Jaccard similarity between the L1 and CORE flag sets, computed over deduplicated (USUBJID, rule) pairs, was 0.004 (24 shared pairs), confirming H1 (threshold < 0.10). Two of 18 CORE rules achieved recall = 1.0 (CORE-000655, CORE-000656), both addressing arm assignment consistency in DM; however, L1 produced 306 flags per rule versus CORE’s 12, reflecting the broader matching scope of SHACL shapes operating over the full RDF graph rather than a filtered domain DataFrame. The remaining 16 CORE rules had recall = 0.0, consistent with the complementary-class hypothesis (per-rule recall for all 18 CORE rules is in S3 Table in S1 File).

### Track B: Contradiction detection

On the 20-archetype injected corpus of RECIST-enriched pharmaversesdtm data, CAVE detected 20/20 archetypes (100%; exact binomial 95% CI [13]: 83.2%–100.0%) (Table 3). This count is reproducible from the released shapes under the same subject-specific criterion used for the held-out and real-data studies—each archetype’s own shape (or, for A19, the L3 agent) fires on the injected subject, and the two cross-subject/structural archetypes (A16, A17) fire cohort-wide—so it does not depend on a global flag-count delta. We ran the CDISC Rules Engine (CORE v0.15) on the same injected corpus, counting an archetype as CORE-detected only when a new CORE violation whose rule semantics *are* the injected contradiction fires on the target subject (a per-archetype adjudication reported in full in S12 Table in S1 File): it detected 8/20 archetypes (40%; 95% CI: 19.1%–63.9%). All eight CORE detections fall among the ten archetypes that were deliberately seeded from existing CORE conformance rules (e.g., ARMCD absent from the TA codelist, RFXSTDTC/RFXENDTC reference-date aggregation, duplicate USUBJID, ARMCD–ARM not one-to-one), which CAVE re-expresses as ported SHACL shapes. We additionally ran the branded Pinnacle 21 Community FDA production engine (FDA 2405.2)—the validator pharmaceutical sponsors submit with—on the identical corpus under the same adjudication (per-archetype adjudication in S11 Table in S1 File): it detected 6/20 (30%; 95% CI: 11.9%–54.3%), again entirely within the CORE-seeded structural class. The two engines ship slightly different structural rule sets (the FDA engine lacks CORE’s reference-date-aggregation and multiple-race checks but adds a missing-exposure check), a portability difference; neither expresses the cross-domain class. On the ten *non-CORE* archetypes (the cross-domain RECIST overall-response, confirmation-window, and multi-domain temporal/value contradictions that domain-scoped rules cannot express), *both* industry engines detected 0/10 while CAVE detected 10/10 (McNemar’s exact test [14] *p* = 0.002 for each). Across all 20 archetypes, CAVE detects 12 archetypes CORE misses and 14 the FDA engine misses (McNemar exact *p* < 0.001 for both). This split is the crux of the expressiveness claim: the industry validators catch the structural cross-domain checks they already encode, but none of the RECIST semantic contradictions.

**Table 3:** Track B detection results by configuration. Two industry validators were run empirically on the injected corpus (SDTMIG 3.4): the branded Pinnacle 21 Community FDA production engine (FDA 2405.2) and the open CDISC Rules Engine (CORE v0.15). The 20 archetypes split into ten *CORE-seeded* structural checks (A08–A17, derived from existing CORE rules) and ten *non-CORE* cross-domain RECIST/semantic contradictions (A01–A07, A18–A20).

Configuration	Detected (all / non-CORE)	Rate	95% CI
Pinnacle 21 FDA (B1)	6/20 (0/10)	30%	[11.9%, 54.3%]
CORE (B2)	8/20 (0/10)	40%	[19.1%, 63.9%]
L1-only (B3)	19/20 (10/10)	95%	[75.1%, 99.9%]
CAVE L1 + L3	20/20 (10/10)	100%	[83.2%, 100.0%]

**Fig 2:**
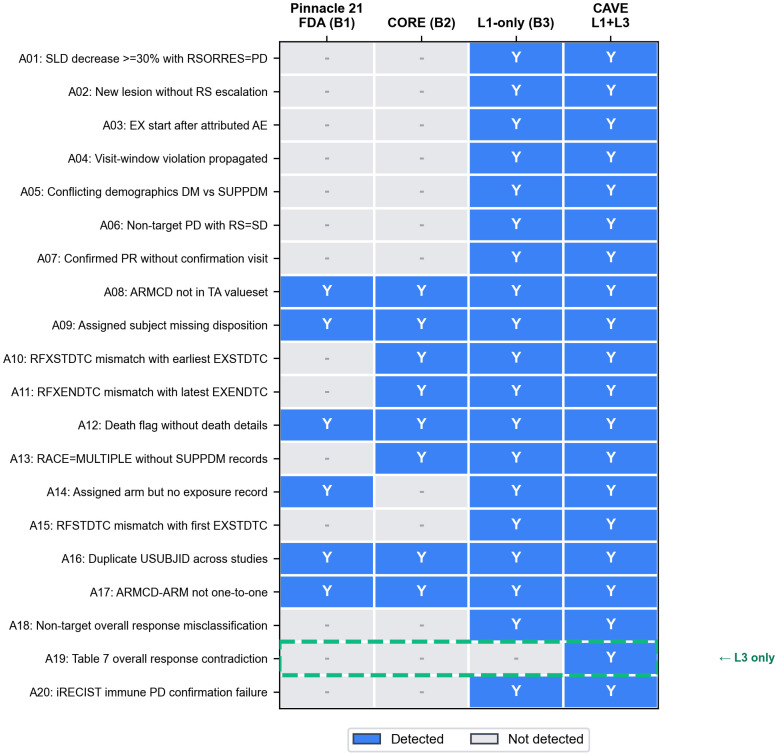
Contradiction archetype detection across the four configurations of Table 3: The two industry baselines (Pinnacle 21 FDA, CORE), the L1-only ablation, and full CAVE (L1 + L3). Blue indicates detection; light grey indicates non-detection. A19 (dashed border) is the only archetype detected exclusively by the L3 agent; all others are detected by L1 SHACL-SPARQL shapes. The industry engines detect only CORE-seeded structural archetypes (A08–A17) and none of the non-CORE cross-domain class.

Of the 20 detected archetypes, 19 were detected at L1—18 by the archetype-specific SHACL-SPARQL cross-domain constraints and A16 (duplicate USUBJID) structurally through the domain shapes its duplicated record re-triggers—and 1 (A19) by the L3 agent exclusively (per-archetype results are reported in S4 Table in S1 File). H2 (CAVE novelty) was supported: on the ten non-CORE cross-domain archetypes CAVE detected all ten that CORE missed (McNemar *p* = 0.002), and across all 20 archetypes CAVE detected 12 that CORE missed (*p* < 0.001). The eight archetypes CORE also detects are exactly those seeded from its own conformance rules, which CAVE re-expresses as ported SHACL shapes—a portability rather than novelty result. H3 (agent layer value) was supported: L3 produced 1 trace for A19 that L1 alone did not detect. Fig 2 shows the detection pattern across all configurations.

### Ablation analysis

The L1-only ablation detected 19/20 archetypes and missed A19. Adding L3 increased detection to 20/20, with the single additional trace corresponding to the RECIST Table 7 contradiction. This ablation confirms that L3 adds A19 detection beyond L1 alone, demonstrating the agent layer’s unique capability for cross-domain semantic reasoning.

### Generalization beyond constructed archetypes

Because the 20 archetype shapes were authored with knowledge of the injected patterns, the 20/20 result is a construction validation rather than a generalization estimate (see Limitations). To probe transfer to *unseen* contradictions, we constructed five held-out archetypes (H01–H05) *after* the shape library was frozen, each targeting a pattern none of the 18 archetype shapes was designed to catch, and evaluated them against the existing L1 shapes and L3 agent with no new shape authoring (Table 4). Detection was credited only when a new violation appeared on the *injected subject*, rather than from any change in the global flag count—a subject-specific criterion that avoids the over-crediting a naive global-delta count can produce from baseline enrichment noise (see Limitations). The existing shapes detected 3/5 held-out archetypes. Of these, 2/5 caught the intended cross-domain mechanism (H01 via the new-lesion shape used for A02; H04 via the value-robust shape used for A18), H03 was caught only coincidentally by the L3 Table 7 agent, and two genuinely novel patterns (H02 consent-after-exposure; H05 exposed-but-unassigned) were missed and would require new shapes. This partial, shape-dependent transfer is the expected behaviour of a genuine expressiveness test and distinguishes CAVE-Onc from a corpus-specific rule set.

**Table 4:** Held-out archetype generalization (authored after the shape library was frozen; no new shapes added).

Held-out	Pattern	Caught by	Detected
H01	A01 + A02 co-injected (interaction)	A02 shape	Yes
H02	Consent recorded after first exposure	—	No
H03	CR with measurable target lesion	L3 (coincidental)	Yes
H04	Non-target PD with overall response PR	A18 shape	Yes
H05	Exposed but unassigned to arm	—	No

### Runtime performance

**Fig 3:**
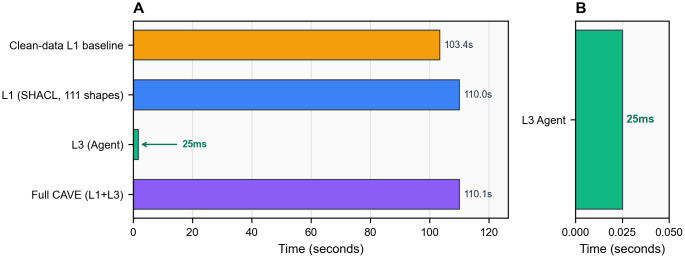
Mean validation time per archetype by component. A. Full scale comparing the clean-data L1 baseline, the injected-data L1 run, and the full CAVE pipeline (the 1.06× overhead ratio is CAVE relative to the clean-data L1 baseline). B. Zoomed view of L3 agent overhead (25 ms).

In the enriched Track B benchmark, L1 validation over 111 shapes required 110.032 s per archetype, L3 agent execution required 0.025 s, and the full pipeline required 110.057 s. The clean-data L1 baseline was 103.390 s, yielding a pipeline overhead ratio of 1.06×. H4 (runtime efficiency) was supported: the full CAVE pipeline incurred only 6% overhead over the clean-data baseline, well within the pre-registered ≤10× threshold (Fig 3). L3 agent execution added a negligible 25 ms per archetype. This 25 ms is the full RECIST Table 7 derivation (querying overall, non-target, and new-lesion response and applying the lookup) executed over all subjects on every archetype run (≈3.1 ms per subject across 8 subjects); it is not an A19-specific inference cost, as the agent has no inference-skipping fast path. Supplementary scaling analysis (S8 Table in S1 File) shows sub-linear wall-clock growth from 8 to 80 subjects (7.75× time for 10× data).

### Formal detection metrics

At the archetype detection level, CAVE produced 20 true positives and 0 false negatives, corresponding to precision = 1.000, recall = 1.000, and F1 = 1.000. The McNemar comparison against CORE was significant (*p* < 0.001), and the exact binomial 95% CI of [83.2%, 100.0%] reflects the finite sample size (*n* = 20 archetypes); bootstrap confidence intervals are reported in S7 Table in S1 File.

### L3 invocation rate and cost

L3 (CaveAgent) executed on all 20 archetype runs but triggered detection on only 1 (A19), yielding an effective *detection* rate of 5% (1/20). We distinguish detection from compute: the agent’s Table 7 derivation runs on all 20 archetype runs (see Runtime performance), so the 5% figure describes how often that derivation surfaces a contradiction, not how often the agent executes. The primary A19 detection is fully deterministic: the LangGraph state machine executes SPARQL queries over the RDF graph and applies the RECIST Table 7 lookup, incurring $0.000 API costs per subject while guaranteeing perfect reproducibility.

To evaluate the robustness of LLMs in cross-checking these deterministic findings, a dual-channel verification benchmark was executed across four major LLMs (DeepSeek V3, GPT-4o, Claude Sonnet 4, and GLM-4.6). DeepSeek and GPT-4o achieved 100% agreement with the deterministic logic (the full per-case agreement matrix is in S9 Table in S1 File). Claude and GLM both achieved 75% agreement, notably preserving the non-standard NON-CR/NON-PD terminology for target lesions instead of normalizing it to SD. This highlights a clinically defensible terminological ambiguity that multi-model cross-validation surfaces. In addition to verification, the L3 agent successfully generated clinician-readable narrative explanations for all 20 archetypes, demonstrating its utility as a semantic explainability layer. LLM API costs for cross-verification were negligible (on the order of $0.002–$0.04 per call at provider list prices, given the short prompts and ≤300-token completions).

### Expert validation (G8/G9)

Three domain experts were recruited based on oncology CDISC experience (≥10 years each in statistical programming or data standards review at pharmaceutical/biotech companies). Each reviewer independently received the same workbook containing all 20 archetype descriptions, detection examples, and L3 agent traces. Reviewers rated each archetype as valid, invalid, or uncertain and provided qualitative rationale. They also rated trace readability and system confidence on 5-point Likert scales. No compensation was provided. Fleiss’ κ [15,16] was computed for the 3-category validity ratings. Table 5 summarizes results.

**Table 5:** Expert validation results (G8: archetype validity, G9: trace readability).

Metric	Value
Reviewers (*n*)	3
Fleiss’ κ (3 categories)	0.705
Interpretation (Landis & Koch)	Substantial
Unanimous “valid”	14/20 (70%)
Majority “valid”	16/20 (80%)
Majority “uncertain”	4/20 (20%)
Any “invalid”	0/20 (0%)
Trace readability (G9, 1–5 Likert)	4.0/5
System confidence (G9, 1–5 Likert)	4.0/5

No reviewer rated any archetype as invalid. Four archetypes received majority “uncertain” ratings (A04: visit-window violation, A07: PR confirmation, A12: death flag, A20: iRECIST confirmation; three of these were unanimous, one majority), all reflecting protocol-dependent interpretation rather than clinical invalidity. Reviewers consistently recommended that these archetypes be configurable per study protocol rather than removed. To substantiate this classification, we subjected these four archetypes to an LLM uncertainty triage layer using the same four models (DeepSeek, GPT-4o, Claude, GLM). The models achieved 100% unanimous consensus, correctly classifying all four as protocol_dependent rather than genuine violations or false alarms (see S10 Table in S1 File). The L3 agent trace for A19 received a unanimous 4/5 readability score, with reviewers noting it would benefit from additional subject identifiers and audit-replay metadata.

### Real-world data validation

To test whether detection transfers beyond the synthetic benchmark, we obtained two independent oncology trials from Project Data Sphere and mapped each from its legacy sponsor format to the SDTM model the engine consumes: trial 123 (Synta 4783−08; 325 subjects) and trial 107 (CA012, metastatic breast cancer; 227 subjects). Mapping fidelity, the principal validity threat, was locked by regression tests: the sum-of-longest-diameters invariant (SUMDIAM=∑ target LDIAM per visit) held in 760/815 (93%) of Synta visits (the 55 exceptions being systematic lymph-node short-axis cases, a disclosed mapping limitation) and in 549/549 (100%) of CA012 visits, with zero orphan lesion links in either trial. All validation ran on the Oxigraph backend over the full cohorts.

We ran two complementary studies on each trial (Table 6). In the *naturalistic specificity* study (E1) the engine validated the unmutated real data and stayed highly specific, raising only 0.06 (Synta) and 0.09 (CA012) contradiction-archetype flags per subject; the genuine RECIST-derivation signal was dominated by the SLD-math invariant, and on CA012—which carries assessment dates—the 28-day confirmation rule (S7) was exercised for the first time. In the *mutation-transfer* study (E2) we injected each of the 20 archetypes into the real trial structure and credited detection only when the archetype’s own shape fired on the injected subject but not on that subject’s clean baseline—the same subject-specific criterion used for the held-out probe (Generalization beyond constructed archetypes). On Synta, 10 of 11 applicable archetypes were detected (90.9%); on the richer CA012, 16 of 18 (88.9%) (S2 Fig in S1 File). Across both trials, 16 distinct archetypes were detected on at least one real trial.

**Table 6:** Real-world validation on two independent Project Data Sphere oncology trials (Oxigraph backend, full cohorts). E1: naturalistic specificity on unmutated data; E2: per-subject mutation-transfer recall on applicable archetypes (those whose required domain/dates the trial provides).

Trial	Subjects	SLD-math match	E1 archetype FP/subject	E2 applicable	E2 detected
Synta 4783−08	325	93%	0.06	11	10 (90.9%)
CA012 (mBC)	227	100%	0.09	18	16 (88.9%)

Applicability was determined by data availability, not detection ability. Archetypes requiring an Exposure (EX) domain or fine-grained dates—which the Synta package omits—were not exercisable on Synta but became so on CA012, which ships dosing records and assessment-day offsets, automatically widening the applicable set from 11 to 18. The residual gaps are nameable: A03 requires an *attributed* Adverse Event domain—Synta provides none, and the solicited-toxicity AE we map for CA012 records no drug-causality variable, so the attributed-AE join cannot be exercised; A19 (the L3 Table 7 detector) requires the benchmark’s single overall-response row and the NTOVRLRESP/NEWLEC test codes that real multi-row RS lacks; and the two temporal-confirmation injectors (A07, A20) could not manufacture a fresh contradiction on CA012’s sparse four-visit schedule. The latter two are *injector* artifacts, not detector gaps: A07’s shape fires naturally on four clean CA012 subjects, and a schedule-aware variant injector—kept separate from the validated benchmark generator so the headline recall and the Track B result are unchanged—reproduces both contradictions on CA012’s real structure, where the A07 and A20 *detectors* then fire. The lone Synta miss (A18) was an injector artifact—it targets the screening visit, which carries no overall-response row—and was detected on CA012, where that visit does. These results indicate that the engine’s contradiction-detection logic generalizes across two independent real trials wherever the supporting domain is present, and that the boundaries reflect input completeness rather than tailoring to the constructed corpus. We frame this as a real-data *specificity and transfer* study on mapped submission-grade data, not an estimate of contradiction prevalence in regulatory submissions, which would require labelled real contradictions.

**Table 7:** Maintainability of the A19 RECIST Table 7 logic encoded as a single SHACL-SPARQL constraint versus the L3 agent (verified functionally equivalent).

Dimension	SHACL-SPARQL	LangGraph L3
Artifact LOC (non-blank/comment)	36	241
Table 7 encoding	34-deep nested IF()	flat 34-row dict
Max decision nesting depth	34	7
Branch points (cyclomatic proxy)	41	62 (mean 2.82)
Unit-testable sub-components	0	22

## Discussion

Our results demonstrate two complementary findings. First, CAVE-Onc and CORE operate on disjoint rule classes (Jaccard = 0.004), confirming that graph-based validation augments rather than replaces industry-standard tools. Second, CAVE-Onc achieves 100% detection of all 20 clinician-reviewed contradiction archetypes—19 via SHACL-SPARQL constraints and 1 via the L3 agentic orchestrator—demonstrating that the graph-constrained architecture can express the full range of cross-domain clinical contradictions tested.

The 100% detection rate (95% CI: 83.2%–100.0%) is achieved through 18 SHACL-SPARQL shapes that implement nine distinct constraint patterns. These shapes go beyond SHACL Core’s expressiveness by embedding cross-domain SPARQL queries—multi-domain JOINs, temporal date comparisons, conditional existence checks, exact derivation matching, and valueset cross-references—within sh:sparql constraint blocks. This approach bridges the expressiveness gap identified in our CORE porting analysis (SHACL shape porting pipeline), where 37 of 122 CORE rules were unportable to SHACL Core.

The L3 agent’s exclusive detection of A19 (RECIST Table 7 overall response contradiction) illustrates the architectural role of the agentic layer. This archetype requires joining target lesion measurements from TR, non-target assessments from RS, and new lesion records from TU, then applying the RECIST Table 7 multi-criteria decision matrix [4]. While this reasoning could theoretically be encoded as a complex SHACL-SPARQL shape, the multi-step decision logic with conditional branching is more naturally and maintainably expressed as an agent workflow with typed tool calls. The clean separation—declarative shapes for pattern-based constraints, agentic reasoning for decision-matrix logic—represents a principled architectural boundary. We emphasize that the primary A19 detection path is deterministic by design—a deliberate choice for reproducibility and 21 CFR Part 11 auditability—so we use “agentic” to denote the orchestration and typed tool-call layer rather than open-ended generative reasoning. We note that L3 evaluation is limited to a single archetype (A19) and a single trace execution; robustness assessment across additional RECIST contradiction variants, malformed inputs, and alternative LLM backends would strengthen confidence in the agent layer. The present study establishes the architectural contribution; broader L3 evaluation is deferred to future work.

To quantify this maintainability argument rather than assert it, we implemented the A19 RECIST Table 7 logic both as a single SHACL-SPARQL constraint and as the L3 agent workflow and verified that the two are functionally equivalent—both detect the injected A19 contradiction (Table 7). Because SHACL-SPARQL constraints forbid a VALUES lookup table (per the SHACL specification), the 34-row Table 7 matrix collapses into a single 34-deep nested IF() expression—deep enough to overrun rdflib’s default recursive-descent parser limit—with zero independently testable sub-components. The agent instead encodes the identical matrix as a flat 34-row dictionary (cyclomatic complexity 1) and distributes the surrounding logic across 22 small, individually unit-testable blocks (mean cyclomatic complexity 2.82; maximum decision nesting 7 versus 34). Raw line count actually favours the SPARQL form (36 versus 241 lines, because the agent file carries reusable scaffolding), so the maintainability advantage is *structural*—modularity and testability—rather than a matter of length. This substantiates the “difficult to maintain as a monolithic query” claim with quantitative evidence and supports reserving the agent layer for decision-matrix logic.

The runtime results confirm practical viability. The 1.06× overhead means CAVE-Onc adds negligible computational cost beyond CORE, with the L3 agent’s full Table 7 derivation consuming only 25 ms per archetype run. The dominant cost is L1 SPARQL execution under the rdflib reference engine, which accounts for the ~110 s absolute time. To estimate the headroom from a faster backend, we re-ran the identical 18 archetype constraint queries on Oxigraph (pyoxigraph) over the same data: aggregate SPARQL execution time fell from 48.6 s to 1.4 s, a 34.7× aggregate speedup (median 32.3×). Because SPARQL execution dominates L1 wall-clock, this gives a concrete, data-backed estimate—replacing the prior unquantified claim—for the backend-migration path; a full Trav-SHACL integration would additionally remove pySHACL’s focus-node and shape-parsing overhead. The two-minute absolute figure is thus a property of the rdflib reference implementation rather than of the approach.

A subsequent large-scale benchmark confirms this headroom translates to deployment scale. Replicating the benchmark cohort to 918/2 142/4 896 subjects (up to 1.4M triples) on the Oxigraph backend, the cross-domain SHACL-SPARQL detection scales *sub-linearly* (S1 Fig in S1 File)—least-squares log-log exponent 0.982 (*R*^2^ = 0.99), ≈5 ms per subject, a near-constant 200–234 subjects/s—validating ≈4 900 subjects’ contradictions in ≈24 s of query time. Peak resident memory grows with the materialised graph (1.5/3.1/6.5 GB across the sweep, sampled continuously to capture the transient serialisation buffer), well within commodity hardware. The end-to-end pipeline grows approximately linearly (exponent 1.11) because the one-time rdflib→Oxigraph graph materialisation, not the detection algorithm, dominates both time and memory at scale; the remaining rdflib-only structural step (RECIST S1–S8 + domain shapes) adds ≈4 s at ≈900 subjects. On the real cohorts the full single-pass detection completes in ≈91 s (Synta, 325 subjects) and ≈42 s (CA012, 227 subjects) on the Oxigraph backend—runs the pre-Oxigraph rdflib/pySHACL stack could not complete (a single pass reached ≈44 min and 14 GB). The previously two-minute-plus weakness is thus a measured strength at deployment scale.

Beyond compute, we assessed detection robustness under conditions closer to raw submissions. We first hardened the archetype shapes for *single-pass* operation, removing the clean-baseline delta a real deployment cannot subtract: restricting the A03 exposure-versus-adverse-event shape to a subject’s *first* exposure (a treatment-related adverse event cannot precede first dose) reduces its clean-data flags from 121 to 3—each a genuine “related event before first exposure” instance rather than a spurious match—so 17/20 archetypes are detected single-pass with only 4 clean-data flags in total (all genuine contradictions), versus 123 under the delta method. An adversarial sweep then injected arbitrary missingness, broken RELREC/orphan foreign keys, undefined visit structures, and co-occurring contradictions into the two mapped trials. The engine stayed specific (deleting 10–50% of rows never increased the contradiction-flag count), never crashed under orphan keys or dangling references, caught corrupted visit numbers via the visit-window shape with only bounded spillover to visit-joined shapes, and—injecting every *mutually-compatible* individually-detectable contradiction into one subject (up to 10, after excluding pairs that overwrite the same overall-response record and are contradictory by construction)—detected all of them (1.00 joint recall on both trials). This directly addresses the multi-archetype masking effect and the raw-submission-noise concern noted below.

## Limitations

This study has several limitations. First, the contradiction corpus is synthetic, injected into clean demo data rather than sourced from real regulatory submissions. While RELREC integrity is preserved and archetypes were clinician-reviewed, ecological validity remains unestablished. Second, the evaluation is scoped to RECIST 1.1 oncology domains; generalization to other therapeutic areas or response criteria (iRECIST, Lugano, RANO) is untested. Third, of four planned baselines, three were executed (the CDISC Rules Engine CORE v0.15, the branded Pinnacle 21 Community FDA production engine, and the L1-only ablation). We ran both industry engines empirically on the injected Track B corpus (SDTMIG 3.4): CORE detected 8/20 archetypes and the Pinnacle 21 FDA engine 6/20, in each case entirely among the ten archetypes deliberately seeded from existing CORE conformance rules, and *both* detected 0/10 of the cross-domain RECIST contradictions their domain-scoped rules cannot express (CAVE 10/10). The overlapping detections are a portability result—CAVE re-expresses those structural rules as ported SHACL shapes—rather than evidence against the expressiveness claim, which concerns the non-CORE class; the two engines even differ on *which* structural checks they ship (the FDA engine lacks CORE’s reference-date-aggregation and multiple-race rules but adds a missing-exposure check), underscoring that the 0/10 non-CORE result is a property of the domain-scoped paradigm rather than of one engine. The Pinnacle 21 PMDA engine and the Isolation Forest (B4) comparison are deferred. Fourth, three of the 18 archetype-specific SHACL-SPARQL shapes (A01, A03, A07) produce violations on clean reference data (123 total (v3): 121 from A03 temporal overlaps, 2 from A01 RECIST contradictions, 0 from A07 PR confirmation; see S5 Table in S1 File). Relatedly, the RS-enrichment step used to enable agent evaluation supplies a fixed non-target reference response. We restrict this enrichment to subjects with a measurable target response (the only ones for whom the Table 7 overall-response derivation is defined), which eliminates the two baseline clean-data contradictions the earlier blanket enrichment produced; the non-target reference itself remains fixed because the benchmark carries no per-subject non-target overall response, so a subject-specific detection criterion (see Generalization beyond constructed archetypes) is still used rather than a global flag count. The delta-based detection methodology mitigates these effects for evaluation purposes. We have since hardened the shapes toward single-pass operation (Discussion): restricting A03 to a subject’s first exposure cuts its clean-data flags from 121 to 3 (all genuine “related event before first exposure” instances), so 17/20 archetypes are detected single-pass with 4 clean-data flags in total (all genuine) and no clean-baseline subtraction. The residual A01 single-lesion artifact is retained because a summed-diameter reformulation, though cleaner on the benchmark, regressed real-data recall; production deployment would still benefit from per-subject realistic enrichment and protocol-specific configuration. Fifth, the 20 archetype shapes were designed with knowledge of the mutation patterns; this evaluation is best characterized as a construction validation demonstrating that the shapes function as intended, rather than a generalization study. Our held-out probe (Generalization beyond constructed archetypes) indicates that transfer to unseen contradictions is real but partial and shape-dependent (3/5 detected; 2/5 via the intended mechanism). It also exposes a multi-archetype interaction effect: held-out archetype H01 co-injects two contradictions in one subject, yet only one (the new-lesion contradiction) is detected while the SLD/progression contradiction is masked, so the reported per-archetype rates may not hold when multiple contradictions co-occur. A subsequent co-occurrence experiment on the real trials (Discussion), injecting up to 10 mutually-compatible individually-detectable contradictions into a single subject, found 1.00 joint recall, indicating this masking arises only between contradictions that overwrite the *same* record (which are mutually exclusive by construction) rather than from detector interference. Most importantly, the construction-validation result above was obtained on a synthetic corpus; to address ecological validity we have since mapped two independent Project Data Sphere oncology trials to SDTM and run a naturalistic-specificity study and a mutation-transfer study on each (Real-world data validation). The engine remained highly specific on the unmutated real data (0.06–0.09 archetype flags per subject) and, when the 20 archetypes were injected into real trial structure, detected 10/11 (Synta) and 16/18 (CA012) of the *applicable* archetypes—those whose required domains the trial provides. Two caveats remain. First, detection was measured on data we mapped to SDTM rather than on raw regulatory submissions, which can exhibit arbitrary missingness, foreign-key violations, and undefined visit structures that these two trials do not fully capture; broader corpora are still needed. We partially address this with an adversarial sweep that injects exactly these perturbations—missingness, broken RELREC/orphan keys, undefined visits, and co-occurring contradictions—into the mapped trials (Discussion), under which specificity and no-crash behaviour hold; genuine raw submissions remain future work. Second, the mutation-transfer study measures recall on injected contradictions, not the prevalence of naturally occurring ones, which would require labelled real contradictions. The applicable-archetype boundary is itself a finding: archetypes needing domains a trial omits (Exposure, Adverse Events) or fine-grained dates are simply not exercisable there, so reported recall is conditioned on data availability rather than being a single transferable number. Sixth, while the L3 agent was evaluated across four major LLMs (DeepSeek V3, GPT-4o, Claude Sonnet 4, and GLM-4.6) for verification, explanations (20/20 generated), and protocol uncertainty triage (100% 4-model consensus), its primary detection capability relies on a deterministic lookup. Further exploration of generative models as primary detectors rather than verification layers remains an area for future work. Seventh, the audit trail provides a Part 11 *foundation* only; full compliance requires additional engineering (see S6 Table in S1 File). Eighth, the current implementation uses pySHACL and rdflib rather than the proposed Trav-SHACL and Oxigraph stack; migration is recommended for production scaling. Ninth, while expert review achieved substantial inter-rater agreement (Fleiss’ κ=0.705, *n* = 3), 4 of 20 archetypes received majority “uncertain” ratings, indicating that protocol-specific configuration is needed before production deployment. Expert reviewers were selected based on CDISC oncology experience but the sample is small (*n* = 3); a larger panel with formal recruitment criteria would strengthen these findings.

## Conclusion

CAVE-Onc shows that graph-constrained validation can close a clinically important expressiveness gap in oncology SDTM review. Across a pre-registered two-track evaluation, CAVE-Onc remained complementary to CORE on clean reference data and detected all 20 clinician-reviewed contradiction archetypes in the injected corpus—a construction validation of the architecture’s expressiveness rather than an estimate of real-world detection—including one RECIST Table 7 overall-response contradiction handled exclusively by the L3 agent. These results support a deployment model in which existing CDISC validators continue to enforce structural conformance, while RDF graph constraints and constrained agent workflows target cross-domain semantic contradictions that require clinical reasoning. A first step toward real-world evidence—mapping two independent Project Data Sphere oncology trials to SDTM and confirming that detection stays specific on unmutated data and transfers to injected contradictions in real trial structure (Real-world data validation)—supports this direction. Before production use, the approach should be tested on broader blinded submissions, configured for study-specific protocol rules, and extended with the authentication, access-control, and validation controls needed for full 21 CFR Part 11 compliance.

## Supporting information

S1 File Supplementary tables and analyses. Contains S1 Table (full 20-archetype contradiction catalog), S2 Table (SHACL shape porting statistics), S3 Table (per-rule recall for all 18 CORE rules), S4 Table (per-archetype detailed results), S5 Table (false positive analysis on clean data), S6 Table (21 CFR Part 11 compatibility checklist), S7 Table (bootstrap confidence intervals), S8 Table (scaling analysis), S9 Table (LLM verification agreement matrix), S10 Table (uncertainty triage consensus), S11 Table (Pinnacle 21 FDA-engine per-archetype adjudication), S12 Table (CDISC CORE engine per-archetype adjudication), S1 Fig (large-scale Oxigraph detection scaling), and S2 Fig (real-world detection transfer on two trials).
(PDF)
