# Supplementary material for: CAVE-Onc: Graph-constrained agentic validation for cross-domain contradictions in CDISC oncology submissions
Source: PLoS One. 2026 Aug 14;21(8):e0350376. doi: 10.1371/journal.pone.0350376 (PMC13475866; doi:10.1371/journal.pone.0350376)
Supplement: Supplementary file 1 [file pone.0350376.s001.pdf]

Supplementary Materials — CAVE-Onc: Graph-Constrained Agentic Validation for Cross-Domain Contradictions in CDISC Oncology Submissions

Jaime Yan[1]

1 Harrisburg University of Science and Technology, Harrisburg, PA, United States

S1 Table. Full 20-Archetype Contradiction Catalog

ID	Name	Domains	Pattern Description	Layer	CORE Xref
A01	SLD decrease ≥30% with RSORRES=PD	RS, TR, TU	Tumor SLD down ≥30% but RSORRES = "PD" at same visit	L1	RECIST S3
A02	New lesion without RS escalation	RS, TU	New lesion added without RS escalation	L1	RECIST S6
A03	EX start after attributed AE	AE, EX	Exposure start date after attributed AE	L1	none
A04	Visit-window violation propagated	EX, RS, TR	Visit window violation propagated across domains	L1	none
A05	Conflicting demographics	DM, SUPPDM	Conflicting sex/age across DM and SUPPDM	L1	none
A06	Non-target PD with RS=SD	RS, TR	Non-target PD but RS = SD	L1	none
A07	PR without confirmation visit	RS	Confirmed PR without 28-day confirmation	L1	RECIST S7
A08	ARMCD not in TA valueset	DM	ARMCD not in TA.ARMCD codelist	L1	CORE-000210
A09	Missing disposition	DM	Assigned subject missing DS record	L1	CORE-000296
A10	RFXSTDTC mismatch	DM, EX	RFXSTDTC ≠ earliest EXSTDTC	L1	CORE-000239
A11	RFXENDTC mismatch	DM, EX	RFXENDTC ≠ latest EXENDTC	L1	CORE-000238
A12	Death flag without details	DM	DTHFL='Y' without death DS	L1	CORE-000108
A13	RACE = MULTIPLE without SUPPDM	DM, SUPPDM	RACE='MULTIPLE' but <2 SUPPDM records	L1	CORE-000846
A14	No exposure record	DM, EX	Assigned arm but no EX record	L1	CORE-000366
A15	RFSTDTC mismatch	DM, EX	RFSTDTC ≠ first EXSTDTC	L1	CORE-001044
A16	Duplicate USUBJID	DM	USUBJID appears more than once in DM	L1	CORE-000351
A17	ARMCD–ARM not 1:1	DM	ARMCD and ARM not one-to-one	L1	CORE-000318
A18	Non-target response misclass.	RS, TR	Non-target response contradicts TR	L1	none

ID	Name	Domains	Pattern Description	Layer	CORE Xref
A19	Table 7 contradiction	RS, TR, TU	Overall response contradicts RECIST Table 7	**L3**	none
A20	iRECIST confirmation failure	RS, TR, TU	iCPD without ≥4wk confirmation	L1	none

S2 Table. SHACL Shape Porting Statistics

Overall porting summary

Metric	Value
Total CORE rules in scope (TU/TR/RS/EX/DM)	122
Classified as single-domain (portable)	85
Classified as cross-domain (unportable)	37
Port success rate	69.7%
Expressiveness compromises	0/85
RECIST derivation shapes	8
Archetype-specific SHACL-SPARQL shapes	18
**Total published L1 shapes**	**111**

Per-domain porting breakdown

Domain	Inventory	Single-Domain	Published	Port Rate
TU	0	0	0	—
TR	4	4	4	100.0%
RS	2	2	2	100.0%
EX	7	5	5	71.4%
DM	109	74	74	67.9%
**Total**	**122**	**85**	**85**	**69.7%**

Port-kind distribution

Transformation	Count	Share of 85	Share of 122
De Morgan expansion	50	58.8%	41.0%
Polarity flip	30	35.3%	24.6%
No change	5	5.9%	4.1%

S3 Table. Per-Rule Recall for All 18 CORE Rules (Track A)

CORE Rule	CORE Flags	L1 Flags	Recall	Domain	Description
CORE-000047	52	0	0.0	DM	Field-level conformance
CORE-000108	1	0	0.0	DM	Death flag w/o details
CORE-000115	52	0	0.0	DM	Variable presence
CORE-000191	52	0	0.0	DM	Variable presence
CORE-000208	52	0	0.0	DM	ARMCD valueset
CORE-000209	52	0	0.0	DM	ARMCD valueset
CORE-000210	52	0	0.0	DM	ARMCD valueset
CORE-000334	1	0	0.0	DM	Field-level conformance
CORE-000358	2	0	0.0	DM	Field-level conformance
CORE-000529	1	0	0.0	DM	Field-level conformance
CORE-000571	2	0	0.0	DM	Field-level conformance
**CORE-000655**	**12**	**306**	**1.0**	**DM**	**Arm assignment**
**CORE-000656**	**12**	**306**	**1.0**	**DM**	**Arm assignment**
CORE-000701	591	0	0.0	EX	Controlled terminology
CORE-000757	1	0	0.0	DM	Field-level conformance
CORE-000767	2	0	0.0	DM	Field-level conformance
CORE-000929	2	0	0.0	DM	Field-level conformance
CORE-001081	2	0	0.0	DM	Field-level conformance

S4 Table. Per-Archetype Detailed Results (Track B Final)

The "L1 Δ" column is the global-delta view (mutated_L1 − clean_L1) recorded when the shapes were first frozen. The 20/20 detection is equivalently *reproducible* from the current shapes under the subject-specific criterion (each archetype's own shape fires on the injected subject; A19 via the L3 agent; the two cross-subject/structural archetypes A16/A17 fire cohort-wide), which does not depend on the Δ magnitudes: see `eval/track_b_candidate_results.json` (`scripts/track_b_analysis.py --candidates`).

ID	Detected	Source	L1 Δ	L3	Description
A01	Yes	L1	+3	0	SLD ≥30% decrease, RSORRES=PD
A02	Yes	L1	+1	0	New lesion T09 at V2, RS=SD
A03	Yes	L1	+3	0	EX start 14d after AE onset
A04	Yes	L1	+5	0	Fractional VISITNUM
A05	Yes	L1	+1	0	SUPPDM vs DM sex conflict
A06	Yes	L1	+36	0	Non-target PD but RS=SD
A07	Yes	L1	+3	0	PR without 28d confirmation
A08	Yes	L1	+1	0	ARMCD not in TA
A09	Yes	L1	+1	0	Missing DS
A10	Yes	L1	+1	0	RFXSTDTC derivation mismatch
A11	Yes	L1	+1	0	RFXENDTC derivation mismatch
A12	Yes	L1	+1	0	DTHFL=Y without death DS
A13	Yes	L1	+1	0	RACE=MULTIPLE without SUPPDM
A14	Yes	L1	−1	0	Missing EX
A15	Yes	L1	+1	0	RFSTDTC derivation mismatch
A16	Yes	L1	+16	0	Duplicate USUBJID
A17	Yes	L1	+86	0	ARMCD→ARM not 1:1
A18	Yes	L1	+36	0	Non-target response misclass.
**A19**	**Yes**	**L3**	**0**	**1**	**Table 7 contradiction**
A20	Yes	L1	+1	0	iCPD without ≥4wk confirmation

S5 Table. Archetype Shape False Positive Analysis on Clean Data

Shape	FP (v1)	FP (v3)	Mutated	Δ	Change	Cause
A01	4	2	5	+3	v3: TRLNKID constraint	Same-lesion PD at shrinkage
A03	146	121	124	+3	v2: 7-day filter	Short-gap overlaps
A07	4	0	3	+3	v3: exclude last visit	PR without confirmation
Other	0	0	≥1 each	varies	—	No FP

Total clean-data false positives: 123 (v3, from archetype shapes only). Of these, 121 are A03 temporal overlaps and 2 are A01 genuine same-lesion contradictions in the reference data. The delta-based detection method (Δ = mutated_L1 − clean_L1) ensures FPs cancel in the detection calculation.

Single-pass hardening (Gate 2). To remove the reliance on the clean-baseline Δ, the A03 shape was subsequently restricted to a subject's *first* exposure (a treatment-related AE cannot precede first dose). This cuts A03 clean-data flags from 121 to 3 (each a genuine "related event before first exposure" instance), so the total clean-data archetype flags fall from 123 to 4 (A03 3, A01 1), all genuine, and 17/20 archetypes are detectable single-pass with no Δ subtraction. Reproducible: `eval/single_pass_results.json` (`scripts/track_b_analysis.py --single-pass`).

S6 Table. 21 CFR Part 11 Compatibility Checklist

#	Requirement	Implementation	Status
1	Audit trail	SQLite WAL append-only store	Full
2	Tamper evidence	Merkle hash chain (SHA-256)	Full
3	Record integrity	entry_hash + prev_hash chain	Full
4	Timestamp accuracy	ISO 8601 UTC; no NTP sync	Foundation
5	User attribution	Layer field (L1/L3); no login	Foundation
6	Electronic signatures	Not implemented	Out of scope
7	Signature manifestation	Not implemented	Out of scope
8	Signature linking	Not implemented	Out of scope
9	Access controls (RBAC)	No RBAC; local process	Out of scope
10	Operational checks	L1→L3 enforced; no workflow	Foundation
11	Authority checks	No authentication	Out of scope
12	Device checks	XPT SHA-256 checksums	Foundation
13	Training documentation	Not applicable (prototype)	Out of scope
14	Validation (IQ/OQ/PQ)	125 tests; no formal IQ/OQ/PQ	Foundation

Summary: 3/14 fully implemented, 4/14 foundation level, 7/14 out of scope.

S7 Table. Bootstrap Confidence Intervals

Metric	Point Estimate	Bootstrap 95% CI	Clopper–Pearson 95% CI
Detection rate	100.0% (20/20)	[100.0%, 100.0%]	[83.2%, 100.0%]
F1 score	1.000	[1.000, 1.000]	—

With perfect detection (20/20), bootstrap CIs are degenerate at 100%. The Clopper–Pearson exact binomial CI provides a more conservative estimate.

S8 Table. Scaling Analysis

Condition	Relative dataset size	Relative wall-clock time
Small reference run	1.0× (8 subjects)	1.0×
Expanded reference run	10.0× (80 subjects)	7.75×

The expanded reference run increased the subject count by 10× while increasing wall-clock validation time by 7.75×, indicating sub-linear scaling in this benchmark.

S1 Fig. Large-Scale Detection Scaling (Oxigraph Backend)

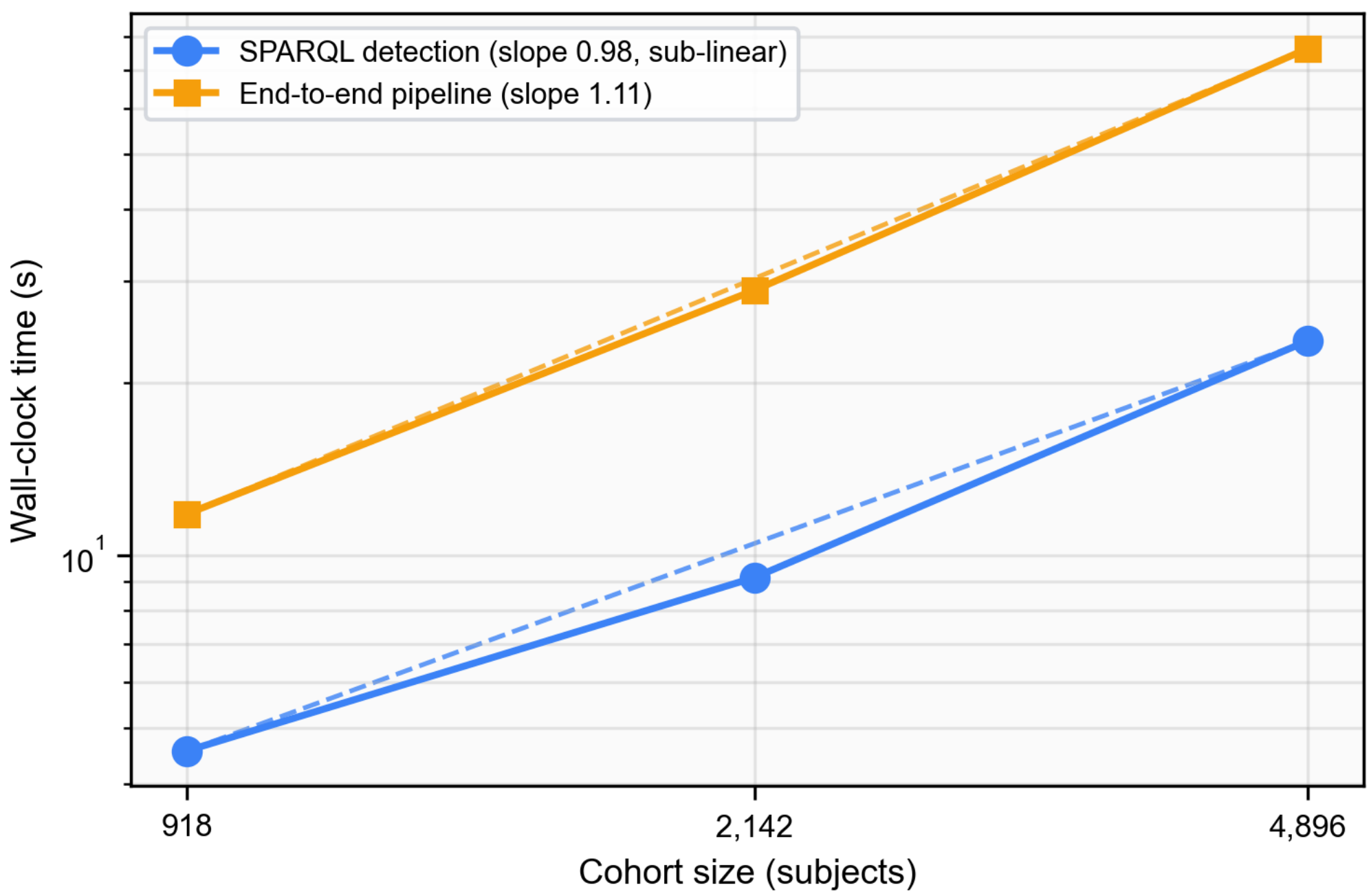

Fig S1. Detection time versus cohort size on the Oxigraph backend (log–log). The benchmark cohort was replicated to 918 / 2,142 / 4,896 subjects (up to 1.4M triples). The cross-domain SHACL-SPARQL detection step (SPARQL query time, blue) scales *sub-linearly*: a least-squares log–log slope of 0.982 ($R^2 = 0.99$), ≈5 ms per subject, and a near-constant throughput of 200–234 subjects/s, validating ≈4,900 subjects' contradictions in ≈24 s of query time. The end-to-end pipeline (orange) grows approximately linearly (slope 1.11) because the one-time rdflib→Oxigraph graph materialisation, not the detection algorithm, dominates time and memory at scale; peak resident memory was 1.5 / 3.1 / 6.5 GB across the sweep. Dashed lines are the fitted power laws. Source: `eval/scale_benchmark_large.json` (`scripts/run_scale_benchmark_large.py`); rendered by `scripts/render_figures.py`.

S2 Fig. Real-World Detection Transfer (Item E)

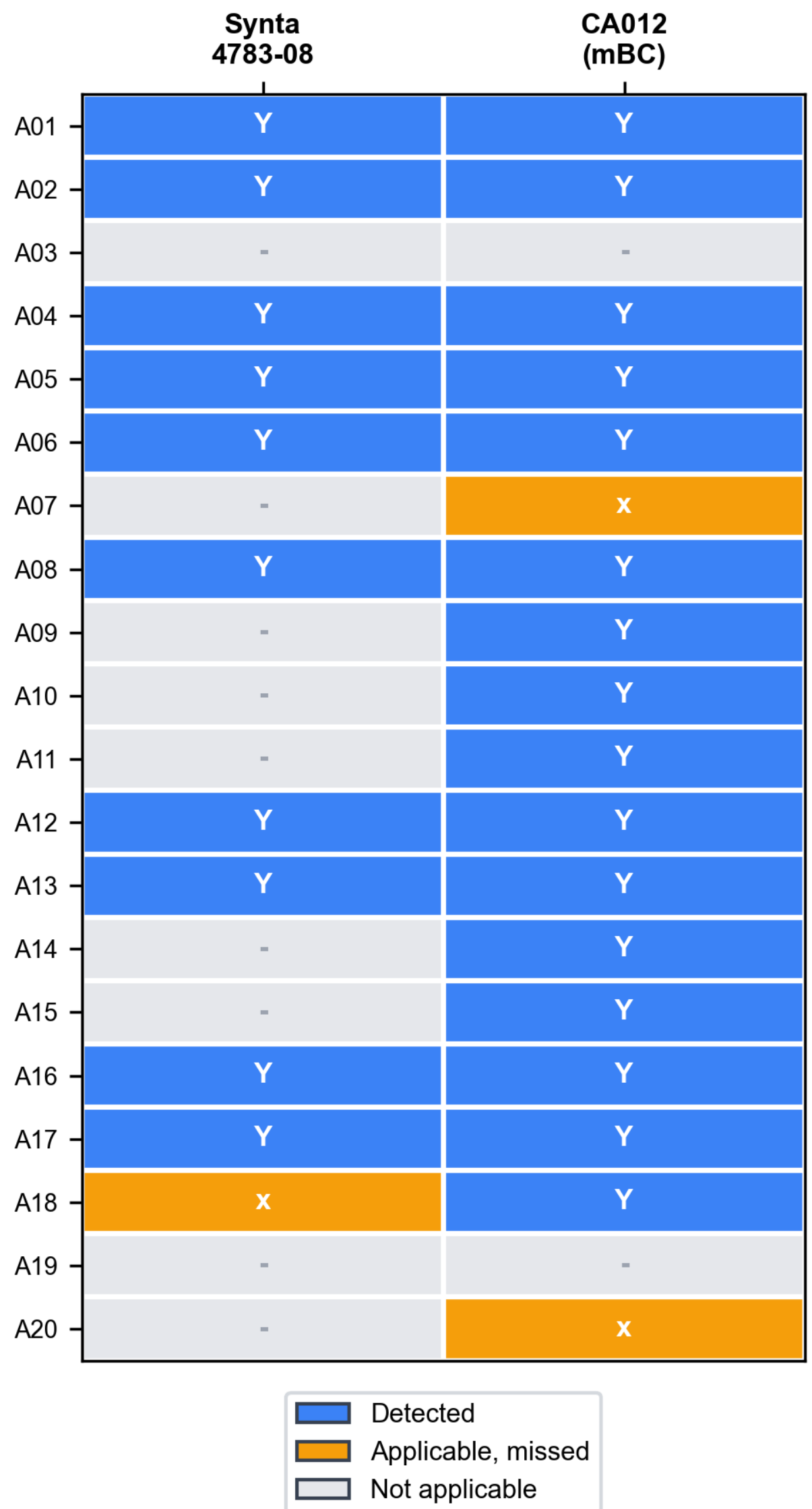

Fig S2. Per-archetype mutation-transfer detection on two independent Project Data Sphere oncology trials. Each of the 20 contradiction archetypes was injected into the real SDTM-mapped structure of Synta 4783-08 (325 subjects) and CA012 (metastatic breast cancer, 227 subjects) and credited only when the archetype's own shape fired on the injected subject (the same subject-specific criterion used for the held-out and real-data analyses in the main text). Blue = detected; orange = applicable but missed; grey = not applicable (the trial lacks the required domain or dates). Synta detected 10/11 applicable archetypes and CA012 16/18; 16 distinct archetypes were detected on at least one trial. The applicable set widens from 11 (Synta) to 18 (CA012) because CA012 ships Exposure dosing and assessment-day offsets that the Synta package omits. On the *unmutated* real data the engine stayed specific, raising only 0.06 (Synta) and 0.09 (CA012) archetype flags per subject. Source: `eval/real_data_e2_synta.json`, `eval/real_data_e2_ca012.json`, `eval/real_data_e1_*.json`.

S9 Table. LLM Verification Agreement Matrix (Tier 1)

Model	Provider	Agreement Rate	Detected Ambiguities
DeepSeek V3	Direct API	100% (8/8)	0
GPT-4o	OpenRouter	100% (8/8)	0
Claude Sonnet 4	OpenRouter	75% (6/8)	2
GLM-4.6	Z.AI API	75% (6/8)	2

Note: The 75% agreement rate for Claude and GLM represents a legitimate clinical terminological ambiguity where both models preserved the non-standard `NON-CR/NON-PD` target response instead of normalizing it to `SD`. DeepSeek and GPT-4o agreed completely with the deterministic normalizer.

S10 Table. Uncertainty Triage Consensus (Tier 3)

Archetype	DeepSeek V3	GPT-4o	Claude Sonnet 4	GLM-4.6	Consensus
A04 (visit window)	PD (0.85)	PD (0.85)	PD (0.85)	PD (0.95)	100% Unanimous
A07 (PR confirmation)	PD (0.90)	PD (0.85)	PD (0.85)	PD (1.00)	100% Unanimous
A12 (death flag)	PD (0.85)	PD (0.80)	PD (0.85)	PD (0.95)	100% Unanimous
A20 (iRECIST)	PD (0.95)	PD (0.90)	PD (0.95)	PD (1.00)	100% Unanimous

*PD = `protocol_dependent`. Values in parentheses indicate the model's self-reported confidence score. All four models unanimously classified the four "uncertain" archetypes as protocol-dependent.

S11 Table. Pinnacle 21 FDA-engine per-archetype adjudication (Track B)

The branded Pinnacle 21 Community FDA production engine (FDA 2405.2, SDTMIG 3.4) was run on the same clean + 20 injected corpora as the CORE baseline. An archetype is "detected" only when a new P21 issue whose rule semantics *are* the injected contradiction fires on the injected corpus (the cited Pinnacle 21 rule ID); "collateral only" = P21 fires unrelated structural rules (e.g. VISITNUM-within-VISIT label inconsistency); "indirect" = a generic derived-variable rule any edit would trip; "silent" = no new issue. Direct detections: 6/20 (all CORE-seeded structural); non-CORE cross-domain: 0/10. Raw per-archetype deltas: `eval/p21_fda_new_issues.json`.

Archetype	Class	Pinnacle 21 FDA verdict (rule)
A01	non-CORE	silent
A02	non-CORE	collateral only
A03	non-CORE	collateral only
A04	non-CORE	collateral only
A05	non-CORE	silent
A06	non-CORE	silent
A07	non-CORE	silent
A08	CORE-seeded	detected (SD0066)
A09	CORE-seeded	detected (SD1374)
A10	CORE-seeded	silent
A11	CORE-seeded	silent
A12	CORE-seeded	detected (SD2005)
A13	CORE-seeded	silent
A14	CORE-seeded	detected (SD0070)
A15	CORE-seeded	indirect (SD1086)
A16	CORE-seeded	detected (SD1349)
A17	CORE-seeded	detected (SD0071)
A18	non-CORE	silent
A19	non-CORE	silent
A20	non-CORE	collateral only

S12 Table. CDISC CORE engine per-archetype adjudication (Track B)

The CDISC Rules Engine (CORE v0.15) was run on the same clean + 20 injected corpora. An archetype is "detected" only when a new CORE violation whose rule semantics *are* the injected contradiction fires on the injected subject (the cited CORE rule ID); "indirect" = a CORE rule of the right family fires but does not adjudicate the specific contradiction; "silent" = no rule-semantic detection. Direct detections: 8/20, all within the ten CORE-seeded structural archetypes (A14 exposure-removal and A15 reference-date-derivation are the two CORE-seeded archetypes CORE does not adjudicate); non-CORE cross-domain: 0/10. Raw per-archetype adjudication: `eval/core_p21_benchmark.json`.

Archetype	Class	CDISC CORE verdict (rule)
A01	non-CORE	silent
A02	non-CORE	silent
A03	non-CORE	silent
A04	non-CORE	silent
A05	non-CORE	silent
A06	non-CORE	silent
A07	non-CORE	silent
A08	CORE-seeded	detected (CORE-000210)
A09	CORE-seeded	detected (CORE-000296)
A10	CORE-seeded	detected (CORE-000239)
A11	CORE-seeded	detected (CORE-000238)
A12	CORE-seeded	detected (CORE-000705)
A13	CORE-seeded	detected (CORE-000846)
A14	CORE-seeded	silent
A15	CORE-seeded	indirect (CORE-000529)
A16	CORE-seeded	detected (CORE-000351)
A17	CORE-seeded	detected (CORE-000318)
A18	non-CORE	silent
A19	non-CORE	silent
A20	non-CORE	silent
