# Supplementary material for: CAVE-Onc: Graph-constrained agentic validation for cross-domain contradictions in CDISC oncology submissions
Source: PLoS One. 2026 Aug 14;21(8):e0350376. doi: 10.1371/journal.pone.0350376 (PMC13475866; doi:10.1371/journal.pone.0350376)
Supplement: Supplementary file 2 [file pone.0350376.s002.docx]

# Response to Reviewers — PONE-D-26-23449

**Title:** CAVE-Onc: Graph-constrained agentic validation for cross-domain contradictions in CDISC oncology submissions

*Final revision (2026-07-07). All revisions are shown in the accompanying tracked-changes manuscript. The new analyses are reproducible via scripts/maintainability_analysis.py, scripts/perf_benchmark.py, and scripts/heldout_analysis.py, with numerical results in eval/maintainability.json, eval/perf_benchmark.json, and eval/heldout_results.json.*

We thank the Academic Editor and both reviewers for their careful and constructive evaluation. The reviews converge on a single, fair central point — that our headline claims outrun a benchmark that is synthetic and, by construction, favorable to the method. We have taken this seriously and have (1) **narrowed the claims** to what the evidence supports — an *expressiveness* contribution rather than a generalization result; (2) **added new quantitative evidence** the reviewers requested (a maintainability comparison, a backend-performance estimate, and a held-out generalization test); and (3) **disclosed more explicitly** the specificity and ecological-validity limitations. We believe these changes materially strengthen the paper while keeping its contribution honestly scoped.

Below we respond to each point. Reviewer text is quoted; our response and the corresponding manuscript changes follow.

## Editor / Journal requirements

*(Numbered to match the editor’s seven requirements.)*

**Requirement 1 (Style / file naming).** *Change:* manuscript and figure files conform to PLOS ONE templates and naming conventions.

**Requirement 2 (Participant consent).** This study uses only **open-source and synthetic data** (CDISC SDTM/ADaM Pilot Project data and the open-source pharmaversesdtm package) and involves **no human subjects, no identifiable patient data, and no new data collection**; informed consent was therefore not applicable and not waived by a committee because no human-subjects research was conducted. (See also Requirement 6 for the full ethics statement.)

**Requirement 3 (Code sharing).** All author-generated code is released without restriction under an OSI-approved license at https://github.com/yanmingyu92/CAVE-Onc-Benchmark. *Change:* the public repository contains the SHACL shapes, the XPT-to-RDF adapter, the L1/L3 engine, the evaluation harness, and the new analysis scripts added in this revision (maintainability, performance, held-out generalization, and the empirical CORE/P21 baseline) together with their result files.

**Requirement 4 (Data Availability — repository name).** *Change:* the Data Availability Statement now names the repository explicitly: “CAVE-Onc-Benchmark (GitHub: yanmingyu92/CAVE-Onc-Benchmark)” and the OSF project (osf.io/fx2ky).

**Requirement 5 (Abstract match).** *Change:* the manuscript abstract was narrowed (per R1-1) to an expressiveness/construction-validation framing, and the submission-form abstract has been updated to match the revised manuscript abstract verbatim (300 words).

**Requirement 6 (Ethics statement in Methods).** *Change:* an explicit “Ethics statement” was added to Methods and the online submission form: “This study analyzed only publicly available, de-identified/synthetic datasets and did not involve human participants; no IRB/ethics-committee review or informed consent was required, as no human-subjects research was conducted.”

**Requirement 7 (Suggested citations).** No specific prior works were mandated; we evaluated the reviewers’ conceptual pointers (e.g., RECIST modelling literature) and cite where relevant.

## Reviewer #1

We thank Reviewer #1 for recognizing the problem as “interesting and practically relevant” and the SHACL + constrained-agent design as “thoughtful” and “potentially useful.” The five concerns below are well taken; our central response is to narrow the claims and add the requested evidence.

### R1-1 — External validity / favorable synthetic benchmark

“The headline result, including 20/20 detection, is obtained on a synthetic contradiction corpus … the 20 archetypes were hand-designed with knowledge of the intended constraint patterns. That makes the benchmark highly favorable …”

**Response.** We agree. We have reframed the contribution throughout from a *detection/generalization* claim to an **expressiveness** claim: CAVE-Onc demonstrates that SHACL-SPARQL graph constraints and a constrained agent *can express* cross-domain oncology contradictions that imperative engines structurally cannot — established here by construction validation. We no longer state or imply that the 100% figure generalizes to real submissions.

To provide evidence *beyond* the constructed corpus, we added two new analyses. First, a **held-out generalization test** (new Results subsection; see R1-2). Second, since the original submission our Project Data Sphere application was granted, and we have now completed a **real-world data validation** on two independent oncology trials (new Results subsection, “Real-world data validation”): we mapped each trial from its legacy sponsor format to SDTM (with fidelity locked by regression tests — the sum-of-diameters invariant holds in 93% and 100% of visits respectively), then ran (i) a naturalistic-specificity study on the unmutated real data (the engine stayed highly specific, 0.06–0.09 archetype flags/subject) and (ii) a mutation-transfer study injecting the 20 archetypes into real trial structure (10/11 and 16/18 of the *applicable* archetypes detected). This directly addresses the ecological-validity concern rather than deferring it.

**Changes.** Abstract and Conclusion narrowed; new “Generalization beyond constructed archetypes” and “Real-world data validation” subsections; Limitations expanded (see R1-2, R1-4, R2-3).

### R1-2 — “Closer to a tailored rule-engine than a blind test of expressiveness”

“19 of the 20 archetypes are essentially encoded directly as bespoke SHACL-SPARQL rules, so the evaluation is closer to a tailored rule-engine demonstration than a blind test of expressiveness.”

**Response.** This is a fair characterization of the original design, which we now label explicitly as *construction validation*. To test genuine generalization, we authored **five held-out archetypes (H01–H05) after the shape library was frozen**, probing patterns the existing 18 shapes were not designed for, and ran them against the **existing** shapes + agent with **no new shape authoring**. Using a rigorous subject-specific detection criterion (a new violation must appear on the *injected subject*), the existing shapes detected **3/5**; only **2/5** caught the *intended* cross-domain mechanism (H01 via the A02 shape; H04 via the A18 shape), while two genuinely novel patterns were missed (H02 consent-after-exposure; H05 exposed-but-unassigned), and one (H03) was caught only coincidentally by the agent. This partial, honest result is exactly what one expects from a real expressiveness test: the constructed shapes transfer to some unseen contradictions and miss others that would require new shapes. We present it as such and have removed any implication of blind completeness.

**Changes.** New Results subsection with the held-out table; Limitations note that detection of unseen patterns is partial and shape-dependent.

### R1-3 — Incomplete baselines (Pinnacle 21 etc.)

“only evaluating against CORE and an L1 ablation is not sufficient … that omission materially weakens the contribution.”

**Response.** We agree, and have now **run the CDISC Rules Engine (CORE v0.15) empirically on the injected Track B corpus** rather than relying on a structural argument. The result is more nuanced than our original “CORE 0/20” and we have corrected the manuscript accordingly. CORE detects **8/20** archetypes — but all eight fall among the **ten archetypes that were deliberately seeded from existing CORE conformance rules** (e.g., ARMCD absent from the TA codelist, RFXSTDTC/RFXENDTC reference-date aggregation, duplicate USUBJID; see the benchmark’s archetype provenance), which CAVE re-expresses as ported SHACL shapes. On the **ten non-CORE archetypes** — the cross-domain RECIST overall-response, confirmation-window, and multi-domain temporal/value contradictions that CORE’s domain-scoped rule language cannot express — CORE detects **0/10** while CAVE detects **10/10** (McNemar exact *p* = 0.002). This empirical split *sharpens* the contribution: the uniquely graph-expressible advantage is precisely the non-CORE class, and the overlap on the eight CORE-seeded archetypes is an honest portability finding, not a novelty one. Regarding Pinnacle 21: we have now also run the **branded Pinnacle 21 Community FDA production engine** (FDA 2405.2) — the validator pharmaceutical sponsors actually submit with — on the identical corpus. It detects **6/20** archetypes, again entirely within the CORE-seeded structural class, and, like CORE, **0/10** of the non-CORE cross-domain contradictions (CAVE 10/10; McNemar exact *p* = 0.002). The two engines even ship slightly different structural rule sets (the FDA engine lacks CORE’s reference-date-aggregation and multiple-race checks but adds a missing-exposure check), which shows the 0/10 non-CORE result is a property of the domain-scoped paradigm, not of one engine. The Pinnacle 21 PMDA engine and the Isolation Forest (B4) baseline remain deferred. Both empirical runs are public and reproducible via scripts/run_p21_fda_baseline.py and scripts/run_core_p21_baseline.sh.

**Changes.** Abstract, Results (Table 3 + text), and Limitations now report **two** empirical industry baselines run on the injected corpus — the open CORE engine (8/20) and the branded Pinnacle 21 FDA production engine (6/20) — both 0/10 vs CAVE 10/10 on the non-CORE cross-domain archetypes; the prior “CORE 0/20” / “P21 wraps CORE” phrasing is replaced. Full adjudication: docs/item_core_p21_findings.md (CORE) and eval/p21_fda_benchmark.json (+ eval/p21_fda_new_issues.json raw deltas; P21).

### R1-4 — Statistics, small sample, and **shapes firing on clean data**

“several SHACL shapes produce violations on clean reference data, which suggests the method currently relies on delta-based suppression or protocol-specific tuning rather than robust specificity. This should be discussed much more explicitly …”

**Response.** We agree and now treat this directly. In the course of this revision we confirmed and quantified the concern: the RS-enrichment step used to enable agent evaluation assigns a fixed non-target response to all subjects, which itself creates baseline RECIST Table 7 contradictions on clean data (two subjects in our corpus). More broadly, three archetype shapes (A01, A03, A07) produce violations on clean reference data, and our held-out analysis motivated a **subject-specific detection criterion** rather than a naive global flag-count delta, which can over-credit detection when baseline enrichment adds noise. We have therefore (1) adopted and reported the subject-specific detection criterion (held-out generalization 3/5), (2) scoped the RS-enrichment to subjects with a measurable target response, which removes the enrichment-induced baseline contradictions, and (3) state plainly that production deployment requires specificity refinement (per-subject realistic enrichment and/or protocol-specific configuration) before the delta-based methodology can be removed. We also retain the appropriately wide exact-binomial interval and present the n=20 result as construction validation, not a powered performance claim.

**Update (post-submission strengthening).** We have since materially reduced the delta reliance itself. Restricting the A03 exposure-versus-adverse-event shape to a subject’s *first* exposure (a treatment-related AE cannot precede first dose) cuts its clean-data flags from 121 to 3 — each a genuine “related event before first exposure” instance rather than a spurious match — so **17/20 archetypes are now detectable single-pass** (one dataset, no clean-baseline subtraction) with only 4 clean-data flags in total, all genuine (eval/single_pass_results.json). The 20/20 Track B result is likewise reproducible under the subject-specific criterion from the current shapes (eval/track_b_candidate_results.json), independent of any global flag-count delta.

**Changes.** Limitations substantially expanded on specificity/delta-suppression; Limitations and the held-out Results subsection note the enrichment-induced baseline contradictions; Results report the rigorous subject-specific criterion; Discussion reports the single-pass hardening (A03 121→3; 17/20 single-pass).

### R1-5 — “Agentic” terminology / L3 necessity

“The Layer 3 ‘agentic’ component is only demonstrated on one archetype (A19) … a largely deterministic workflow rather than a genuinely agentic reasoning system.”

**Response.** We agree the primary A19 path is deterministic by design — a deliberate choice for reproducibility and 21 CFR Part 11 auditability — and we now say so explicitly, reserving “agentic” for the orchestration/typed-tool-call layer rather than implying open-ended reasoning. To substantiate the *necessity* of a separate layer (rather than terminology alone), we add the quantitative maintainability comparison Reviewer #2 requested (see R2-1): encoding the A19 RECIST Table 7 logic as a single SHACL-SPARQL constraint requires a **34-deep nested IF()** expression with **zero independently testable sub-components** (and one so deep it overruns the standard SPARQL parser), whereas the agent expresses the identical, verified-equivalent logic as a flat lookup table plus 22 small, individually testable steps. This supports the architectural boundary — declarative shapes for pattern constraints, a modular agent workflow for decision-matrix logic — on maintainability grounds, independent of the “agentic” label.

**Changes.** Discussion softens the “agentic” framing and adds the maintainability evidence and table; we note L3 evaluation remains limited to A19 (already in Limitations).

## Reviewer #2

We thank Reviewer #2 for finding the motivation clear, the technical layers distinct, and the evaluation design rigorous, and for the specific, actionable requests below.

### R2-1 — Quantify maintainability (line count + branch complexity)

“Could the author provide an example that compares the line count and branch complexity between SHACL-SPARQL coding and LangGraph workflow coding?”

**Response.** Yes. We implemented the A19 RECIST Table 7 logic both ways and verified the two implementations are **functionally equivalent** (both detect the injected A19 contradiction), then measured them (radon for the Python path; query-structure analysis for the SPARQL path):

| Dimension | SHACL-SPARQL (single shape) | LangGraph L3 (agent) |
| --- | --- | --- |
| Artifact LOC (non-blank/comment) | 36 | 241 |
| Table 7 encoding | **34-deep nested IF()** (SHACL forbids VALUES) | **flat 34-row dict** (cyclomatic complexity 1) |
| Max decision nesting | 34 | 7 |
| Branch points | 41 | total CC 62 (avg 2.82, max 7) |
| Independently unit-testable sub-components | **0 (monolithic)** | **22** |

We are careful not to overstate: raw line count actually *favours* the SPARQL form (36 vs 241), because the agent file carries reusable framework scaffolding. The maintainability gap is therefore **structural rather than line-count**: the SPARQL form collapses the matrix, three cross-record joins, three normalizations and four filters into one ~20-line query with a 34-deep nested expression and no isolatable, testable sub-steps; the agent form distributes the same logic into 22 small testable blocks with the matrix as a diff-friendly table. This is the basis for our “difficult to maintain” claim, now quantified.

**Changes.** New maintainability table and paragraph in Discussion (see also R1-5).

### R2-2 — Performance: absolute runtime, backend speedup range, and what 25 ms measures

“the nearly two-minute absolute time per prototype may be excessive … it fails to provide an estimated range of performance improvements. Additionally, is the 25 ms for L3 the inference time exclusive to A19, or the routing overhead … for all prototypes?”

**Response — the 25 ms.** We clarify: the 25 ms is the **full L3 agent execution per archetype run** — querying RS for OVRLRESP/NTOVRLRESP/NEWLEC, applying the Table 7 lookup, and comparing — executed over **all** subjects on **every** run (≈3.1 ms/subject across 8 subjects; the contradiction-emission step is negligible, within measurement noise). It is **not** “routing for 19 archetypes plus inference for A19.” The agent has no inference-skipping fast path; compute is incurred on all 20 runs. The previously reported “5% invocation rate” refers to **detection** (1/20 archetypes yields a trace), not to compute. We have corrected the manuscript wording to distinguish *execution* from *detection/trigger*.

**Response — backend speedup range.** We provide a concrete estimate by benchmarking the **same 18 archetype SPARQL constraint queries** on the current rdflib engine versus Oxigraph (pyoxigraph) over identical data: aggregate execution time falls from 48.6 s to 1.40 s, a **~34.7× aggregate speedup (median 32.3×)**. Since SPARQL execution dominates L1 wall-clock, this gives a data-backed range for the backend-migration claim (replacing the previous unquantified statement). We note this is an engine-level micro-benchmark; a full Trav-SHACL integration would additionally remove pySHACL focus-node and shape-parsing overhead, and we frame the absolute two-minute figure as a property of the rdflib reference implementation rather than the approach.

**Update (post-submission strengthening).** We have since run a **real large-scale benchmark** on the Oxigraph backend at 918 / 2 142 / 4 896 synthetic subjects (scripts/run_scale_benchmark_large.py, eval/scale_benchmark_large.json): the cross-domain SHACL-SPARQL detection scales **sub-linearly** (least-squares log-log exponent 0.982, R²=0.99; ~5 ms/subject; ~200–234 subjects/s), validating ~4 900 subjects’ contradictions in ~24 s of query time, with peak memory 1.5–6.5 GB (continuously sampled). The full real cohorts complete end-to-end in ~91 s (Synta, 325 subjects) and ~42 s (CA012, 227 subjects) — runs the pre-Oxigraph stack could not finish — so the two-minute weakness becomes a measured strength at deployment scale (Discussion).

**Changes.** Results clarify the 25 ms decomposition and the execution-vs-detection distinction; Discussion adds the ~30–35× Oxigraph estimate with the micro-benchmark caveat.

### R2-3 — Synthetic data; missingness, FK violations, multi-archetype interaction

“it remains unclear whether the 100% detection rate … would generalize to real regulatory submission data … It is also suggested that the authors explicitly acknowledge in the limitations section that the method has not been validated on real-world data.”

**Response.** We agree, and we have gone beyond acknowledgement. We now report a **real-world data validation** on two independent Project Data Sphere oncology trials (new Results subsection; see R1-1). On the *unmutated* real data the engine remained highly specific (0.06–0.09 archetype flags per subject), and when the 20 archetypes were injected into real trial structure it detected 10/11 (Synta) and 16/18 (CA012) of the **applicable** archetypes — those whose required domains the trial actually provides. We are deliberately careful not to overclaim: the Limitations section states that this was measured on data we mapped to SDTM rather than on raw regulatory submissions (which can exhibit arbitrary missingness, foreign-key violations, and undefined visit structures these two trials do not fully capture), and that mutation-transfer measures recall on injected contradictions rather than the prevalence of naturally occurring ones. We additionally provide a probe of the **multi-archetype interaction** case: held-out archetype H01 co-injects two contradictions in one subject, and we observe **partial masking** — only the new-lesion contradiction is detected while the SLD/PD contradiction is missed in the multi-archetype context, concretely illustrating the interaction effect the reviewer raised.

**Update (post-submission strengthening).** We have since addressed the reviewer’s three specific perturbation concerns *directly*, rather than only acknowledging them. An adversarial robustness sweep (scripts/run_robustness_sweep.py, eval/robustness_sweep.json) injects exactly these perturbations — arbitrary missingness (10–50% row deletion), broken RELREC / orphan foreign keys, undefined visit structures (corrupted VISITNUM), and co-occurring contradictions — into the two mapped trials. The engine (i) stays specific under missingness (deleting rows never increases the contradiction-flag count), (ii) never crashes under orphan keys or dangling references, (iii) catches corrupted visits via the visit-window shape with only bounded spillover, and (iv) refines the multi-archetype finding: injecting every *mutually-compatible* individually-detectable contradiction into one subject (up to 10) yields **1.00 joint recall**, showing the H01 masking arises specifically between contradictions that overwrite the *same* overall-response record (mutually exclusive by construction) rather than from detector interference. This is reported in the Discussion.

**Changes.** New “Real-world data validation” Results subsection (two PDS trials, E1 specificity + E2 mutation transfer); Limitations updated to report the real-data validation with its remaining caveats; held-out results include the H01 masking finding; Discussion adds the adversarial robustness sweep (missingness / FK / undefined visits / co-occurrence) and the efficiency scale benchmark.

We are grateful for the reviewers’ time and believe the revised manuscript is more rigorous, more honestly scoped, and more useful as a result. We are happy to provide the new analysis scripts and raw outputs to the reviewers on request.
