# Supplementary material for: CAVE-Onc: Graph-constrained agentic validation for cross-domain contradictions in CDISC oncology submissions
Source: PLoS One. 2026 Aug 14;21(8):e0350376. doi: 10.1371/journal.pone.0350376 (PMC13475866; doi:10.1371/journal.pone.0350376)
Supplement: Peer review history

# Peer review: Decision Letter 0

**DOI:** 10.1371/journal.pone.0350376.r001

**Authors:** Le Zhang

10 Jun 2026

PONE-D-26-23449

CAVE-Onc: Graph-constrained agentic validation for cross-domain contradictions in CDISC oncology submissions

PLOS One

Dear Dr. Yan,

Thank you for submitting your manuscript to PLOS ONE. After careful consideration, we feel that it has merit but does not fully meet PLOS ONE’s publication criteria as it currently stands. Therefore, we invite you to submit a revised version of the manuscript that addresses the points raised during the review process.

Please submit your revised manuscript by Aug 09 2026 11:59PM. If you will need more time than this to complete your revisions, please reply to this message or contact the journal office at plosone@plos.org. When you're ready to submit your revision, log on to https://www.editorialmanager.com/pone/ and select the 'Submissions Needing Revision' folder to locate your manuscript file.

Please include the following items when submitting your revised manuscript:

- A letter that responds to each point raised by the academic editor and reviewer(s). You should upload this letter as a separate file labeled 'Response to Reviewers'.
- A marked-up copy of your manuscript that highlights changes made to the original version. You should upload this as a separate file labeled 'Revised Manuscript with Track Changes'.
- An unmarked version of your revised paper without tracked changes. You should upload this as a separate file labeled 'Manuscript'.

If you would like to make changes to your financial disclosure, please include your updated statement in your cover letter. Guidelines for resubmitting your figure files are available below the reviewer comments at the end of this letter.

If applicable, we recommend that you deposit your laboratory protocols in protocols.io to enhance the reproducibility of your results. Protocols.io assigns your protocol its own identifier (DOI) so that it can be cited independently in the future. For instructions see: https://journals.plos.org/plosone/s/submission-guidelines#loc-laboratory-protocols. Additionally, PLOS ONE offers an option for publishing peer-reviewed Lab Protocol articles, which describe protocols hosted on protocols.io. Read more information on sharing protocols at https://plos.org/protocols?utm_medium=editorial-email&utm_source=authorletters&utm_campaign=protocols.

As the corresponding author, your ORCID iD is verified in the submission system and will appear in the published article. PLOS supports the use of ORCID, and we encourage all coauthors to register for an ORCID iD and use it as well. Please encourage your coauthors to verify their ORCID iD within the submission system before final acceptance, as unverified ORCID iDs will not appear in the published article. *Only*  the individual author can complete the verification step; PLOS staff *cannot*  verify ORCID iDs on behalf of authors.

We look forward to receiving your revised manuscript.

Kind regards,

Le Zhang

Academic Editor

PLOS One

**Journal Requirements:**

1. When submitting your revision, we need you to address these additional requirements.

Please ensure that your manuscript meets PLOS ONE's style requirements, including those for file naming. The PLOS ONE style templates can be found at

https://journals.plos.org/plosone/s/file?id=wjVg/PLOSOne_formatting_sample_main_body.pdf and

https://journals.plos.org/plosone/s/file?id=ba62/PLOSOne_formatting_sample_title_authors_affiliations.pdf

2. Please provide additional details regarding participant consent. In the ethics statement in the Methods and online submission information, please ensure that you have specified (a) whether consent was informed and (b) what type you obtained (for instance, written or verbal, and if verbal, how it was documented and witnessed). If your study included minors, state whether you obtained consent from parents or guardians. If the need for consent was waived by the ethics committee, please include this information.

If you are reporting a retrospective study of medical records or archived samples, please ensure that you have discussed whether all data were fully anonymized before you accessed them and/or whether the IRB or ethics committee waived the requirement for informed consent. If patients provided informed written consent to have data from their medical records used in research, please include this information.

3. Please note that PLOS One has specific guidelines on code sharing for submissions in which author-generated code underpins the findings in the manuscript. In these cases, we expect all author-generated code to be made available without restrictions upon publication of the work. Please review our guidelines at https://journals.plos.org/plosone/s/materials-and-software-sharing#loc-sharing-code and ensure that your code is shared in a way that follows best practice and facilitates reproducibility and reuse.

4. Please note that your Data Availability Statement is currently missing the repository name. If your manuscript is accepted for publication, you will be asked to provide these details on a very short timeline. We therefore suggest that you provide this information now, though we will not hold up the peer review process if you are unable.

5. Please amend either the abstract on the online submission form (via Edit Submission) or the abstract in the manuscript so that they are identical.

6. Please include your full ethics statement in the ‘Methods’ section of your manuscript file. In your statement, please include the full name of the IRB or ethics committee who approved or waived your study, as well as whether or not you obtained informed written or verbal consent. If consent was waived for your study, please include this information in your statement as well.

7. If the reviewer comments include a recommendation to cite specific previously published works, please review and evaluate these publications to determine whether they are relevant and should be cited. There is no requirement to cite these works unless the editor has indicated otherwise.

[Note: HTML markup is below. Please do not edit.]

Reviewers' comments:

Reviewer's Responses to Questions

**Comments to the Author**

1. Is the manuscript technically sound, and do the data support the conclusions?

The manuscript must describe a technically sound piece of scientific research with data that supports the conclusions. Experiments must have been conducted rigorously, with appropriate controls, replication, and sample sizes. The conclusions must be drawn appropriately based on the data presented.

Reviewer #1: Partly

Reviewer #2: Yes

**********

2. Has the statistical analysis been performed appropriately and rigorously?

Reviewer #1: Yes

Reviewer #2: Yes

**********

3. Have the authors made all data underlying the findings in their manuscript fully available?

The PLOS Data policy requires authors to make all data underlying the findings described in their manuscript fully available without restriction, with rare exception (please refer to the Data Availability Statement in the manuscript PDF file). The data should be provided as part of the manuscript or its supporting information, or deposited to a public repository. For example, in addition to summary statistics, the data points behind means, medians and variance measures should be available. If there are restrictions on publicly sharing data—e.g. participant privacy or use of data from a third party—those must be specified.

Reviewer #1: Yes

Reviewer #2: Yes

**********

4. Is the manuscript presented in an intelligible fashion and written in standard English?

PLOS ONE does not copyedit accepted manuscripts, so the language in submitted articles must be clear, correct, and unambiguous. Any typographical or grammatical errors should be corrected at revision, so please note any specific errors here.

Reviewer #1: Yes

Reviewer #2: Yes

**********

5. Review Comments to the Author

Please use the space provided to explain your answers to the questions above. You may also include additional comments for the author, including concerns about dual publication, research ethics, or publication ethics. (Please upload your review as an attachment if it exceeds 20,000 characters)

**Reviewer #1:**  This manuscript addresses an interesting and practically relevant problem: extending CDISC validation beyond rule-based checks to capture cross-domain semantic contradictions in oncology submissions. The system design is thoughtful, and the idea of combining SHACL with a constrained agentic layer is potentially useful. However, in its current form, the evidence is not yet strong enough to support the broad claims made in the abstract and conclusion.

My main concern is external validity. The headline result, including 20/20 detection, is obtained on a synthetic contradiction corpus that was injected into clean open-source datasets, and the 20 archetypes were hand-designed with knowledge of the intended constraint patterns. That makes the benchmark highly favorable to the proposed system and limits the strength of the claim that the method generalizes to real regulatory submissions. In addition, 19 of the 20 archetypes are essentially encoded directly as bespoke SHACL-SPARQL rules, so the evaluation is closer to a tailored rule-engine demonstration than a blind test of expressiveness.

The comparison against existing tools is also incomplete. For a manuscript making a practical validation claim, only evaluating against CORE and an L1 ablation is not sufficient. The manuscript notes that Pinnacle 21 and other planned baselines were not functional, but that omission materially weakens the contribution. Without a stronger set of accepted baselines, it is hard to judge whether the proposed framework is genuinely better, or simply differently engineered.

The statistical treatment is technically correct in places, but not fully convincing. A sample of 20 archetypes is very small for strong performance claims, and the exact binomial CI is necessarily wide. The McNemar test on a hand-crafted injected corpus is also not very informative about real-world utility. I was also concerned that several SHACL shapes produce violations on clean reference data, which suggests the method currently relies on delta-based suppression or protocol-specific tuning rather than robust specificity. This should be discussed much more explicitly, because it directly affects deployability.

The Layer 3 “agentic” component is only demonstrated on one archetype (A19), and the description suggests a largely deterministic workflow rather than a genuinely agentic reasoning system. The manuscript should either justify this terminology more carefully or simplify the framing. As written, the necessity of the extra layer is only weakly supported.

The writing is generally understandable, though some claims are overstated relative to the evaluation design, especially in the abstract and conclusion. I would recommend a substantial revision that narrows the claims, adds stronger external baselines, and provides validation on real or held-out regulatory data before drawing broad conclusions.

**Reviewer #2:**  This paper proposes CAVE-Onc, a two-layer graph-constraint verification framework for detecting cross-domain semantic inconsistencies in oncology SDTM data. This work has clear practical motivations, distinct technical layers, and a rigorous evaluation design. Experiments demonstrate that CAVE is highly complementary to CORE and can detect all 20 predefined contradiction prototypes, with substantial expert agreement. The overall quality is satisfactory for publication. However, revisions are required in the following aspects to improve academic standardization and readability.

1.Only A19 was uniquely detected by L3 (Overall Response Discrepancy in RECIST Table 7). The paper acknowledges that "this logic can also be encoded as complex SHACL-SPARQL, yet it is difficult to maintain". Nevertheless, the difference in maintainability has not been verified through user experiments or quantitative metrics such as rule-writing time and cognitive load. Could the author provide an example that compares the line count and branch complexity between SHACL-SPARQL coding and LangGraph workflow coding?

2.Validating each prototype with L1 takes 110 seconds (Track B), while the CORE baseline records 103.4 seconds (Fig 3). Despite the relatively small overhead, the nearly two-minute absolute time per prototype may be excessive for real-world submissions involving hundreds of subjects. The paper mentions that migration to Trav-SHACL or Oxigraph can bring optimizations, yet it fails to provide an estimated range of performance improvements. Additionally, is the 25ms for L3 the inference time exclusive to A19, or the routing overhead incurred by L3 for all prototypes? The article states "L3 invocation rate = 5% (1/20)". If L3 only performs core inference on A19, is the 25 ms allocated to the remaining 19 prototypes solely used for routing judgment?

3.The contradictory corpora in this study are synthetically injected rather than sourced from real regulatory submission data. While synthetic data boasts the advantages of controllability and reproducibility, it fails to capture the complexity of real-world scenarios, including arbitrary patterns of missing data, foreign key relationships that violate assumptions, undefined subject-visit structures, and interaction effects when multiple contradictions coexist. Therefore, it remains unclear whether the 100% detection rate reported on synthetic data would generalize to real regulatory submission data, which may involve uncontrolled noise, missingness, and multi-archetype interactions. It is also suggested that the authors explicitly acknowledge in the limitations section that the method has not been validated on real-world data.

**********

6. PLOS authors have the option to publish the peer review history of their article (what does this mean?). If published, this will include your full peer review and any attached files.

If you choose “no”, your identity will remain anonymous but your review may still be made public.

**Do you want your identity to be public for this peer review?**  For information about this choice, including consent withdrawal, please see our Privacy Policy.

Reviewer #1: No

Reviewer #2: No

**********

[NOTE: If reviewer comments were submitted as an attachment file, they will be attached to this email and accessible via the submission site. Please log into your account, locate the manuscript record, and check for the action link "View Attachments". If this link does not appear, there are no attachment files.]

To ensure your figures meet our technical requirements, please review our figure guidelines: https://journals.plos.org/plosone/s/figures

You may also use PLOS’s free figure tool, NAAS, to help you prepare publication quality figures: https://journals.plos.org/plosone/s/figures#loc-tools-for-figure-preparation.

NAAS will assess whether your figures meet our technical requirements by comparing each figure against our figure specifications.

# Author comment: Author response to Decision Letter 1

**DOI:** 10.1371/journal.pone.0350376.r002

16 Jul 2026

Please see the attached "Response to Reviewers" document for our detailed, point-by-point responses and the new analyses.

Attachment Submitted filename: response_to_reviewers.docx

# Editor report: Decision Letter 1

**DOI:** 10.1371/journal.pone.0350376.r003

**Authors:** Le Zhang

2 Aug 2026

CAVE-Onc: Graph-constrained agentic validation for cross-domain contradictions in CDISC oncology submissions

PONE-D-26-23449R1

Dear Dr. Yan,

We’re pleased to inform you that your manuscript has been judged scientifically suitable for publication and will be formally accepted for publication once it meets all outstanding technical requirements.

Within one week, you’ll receive an e-mail detailing the required amendments. When these have been addressed, you’ll receive a formal acceptance letter and your manuscript will be scheduled for publication.

An invoice will be generated when your article is formally accepted. Please note, if your institution has a publishing partnership with PLOS and your article meets the relevant criteria, all or part of your publication costs will be covered. Please make sure your user information is up-to-date by logging into Editorial Manager at Editorial Manager® and clicking the ‘Update My Information' link at the top of the page. For questions related to billing, please contact billing support.

If your institution or institutions have a press office, please notify them about your upcoming paper to help maximize its impact. If they’ll be preparing press materials, please inform our press team as soon as possible -- no later than 48 hours after receiving the formal acceptance. Your manuscript will remain under strict press embargo until 2 pm Eastern Time on the date of publication. For more information, please contact onepress@plos.org.

Kind regards,

Le Zhang

Academic Editor

PLOS One

Additional Editor Comments (optional):

Reviewers' comments:

# Editor report: Acceptance letter

**DOI:** 10.1371/journal.pone.0350376.r004

**Authors:** Le Zhang

PONE-D-26-23449R1

PLOS One

Dear Dr. Yan,

I'm pleased to inform you that your manuscript has been deemed suitable for publication in PLOS One. Congratulations! Your manuscript is now being handed over to our production team.

At this stage, our production department will prepare your paper for publication. This includes ensuring the following:

* All references, tables, and figures are properly cited

* All relevant supporting information is included in the manuscript submission,

* There are no issues that prevent the paper from being properly typeset

You will receive further instructions from the production team, including instructions on how to review your proof when it is ready. Please keep in mind that we are working through a large volume of accepted articles, so please give us a few days to review your paper and let you know the next and final steps.

Lastly, if your institution or institutions have a press office, please let them know about your upcoming paper now to help maximize its impact. If they'll be preparing press materials, please inform our press team within the next 48 hours. Your manuscript will remain under strict press embargo until 2 pm Eastern Time on the date of publication. For more information, please contact onepress@plos.org.

You will receive an invoice from PLOS for your publication fee after your manuscript has reached the completed accept phase. If you receive an email requesting payment before acceptance or for any other service, this may be a phishing scheme. Learn how to identify phishing emails and protect your accounts at https://explore.plos.org/phishing.

If we can help with anything else, please email us at customercare@plos.org.

Thank you for submitting your work to PLOS ONE and supporting open access.

Kind regards,

PLOS ONE Editorial Office Staff

on behalf of

Dr. Le Zhang

Academic Editor

PLOS One
